# Piroxicam accelerates diabetic foot ulcer healing via ERα-dependent mitochondrial protection and oxidative stress relief

**DOI:** 10.3389/fphar.2026.1834818

**Published:** 2026-07-24

**Authors:** Qing-Qing Liao, Li-Ping Chen, Jia-Qi Zheng, Yu-Jie Yang, Jing-Dan Weng, Teng Xie, Xiao-Dong Sun, Yuan-Yuan Zhang

**Affiliations:** 1 Department of Pharmacology, West China School of Pharmacy, West China School of Basic Medical Sciences & Forensic Medicine, Sichuan University, Chengdu, China; 2 Tianfu Jincheng Laboratory, Chengdu, China

**Keywords:** diabetic foot ulcer, estrogen receptor α, inflammation, mitochondrial dysfunction, piroxicam

## Abstract

**Introduction:**

The pathology of diabetic foot ulcer (DFU) is characterized by keratinocyte dysfunction, non-resolving inflammation, and oxidative stress. We aim to investigate the effects and mechanisms of piroxicam on DFU healing through regulating mitochondrial function and suppressing inflammation.

**Methods:**

DFU was established in male C57BL/6 J mice and ovariectomized female mice. Piroxicam (1% or 0.33%) solution or saline was then applied for 9 days. HaCaT cells were induced with high glucose (HG) and subsequently incubated with piroxicam (0, 1.2, 3.7, 11, 33, 100 nM).

**Results and Discussion:**

Piroxicam significantly promoted DFU healing and inhibited the fibrosis in male diabetic mice at a low dose. Consistently, piroxicam enhanced proliferation and migration, and inhibited inflammation, fibrosis, and cellular senescence in HG-induced HaCaT cells. Mechanistically, piroxicam alleviated HG-induced mitochondrial dysfunction by stabilizing the mitochondrial respiratory chain, increasing biogenesis, and enhancing mitophagy. These effects further attenuated oxidative stress and inhibited the cGAS-STING-NF-κB inflammatory pathway, thereby reducing the release of pro-inflammatory factors. Furthermore, molecular docking revealed that piroxicam bound to ERα, a finding further confirmed by a cellular thermal shift assay. HG induced a significant decrease in nuclear ERα protein levels, which was reversed by piroxicam, especially at 11 and 33 nM. Additionally, piroxicam’s pro-healing and anti-inflammation effects were attenuated in ovariectomized female DFU mice. Piroxicam’s protection of mitochondrial function and suppression of oxidative stress was also abolished upon blocking ERα by tamoxifen. In conclusion, piroxicam alleviates mitochondrial dysfunction and suppresses inflammatory responses by binding to ERα, which ultimately promotes DFU healing at low doses.

## Graphical Abstract

**Figure:**
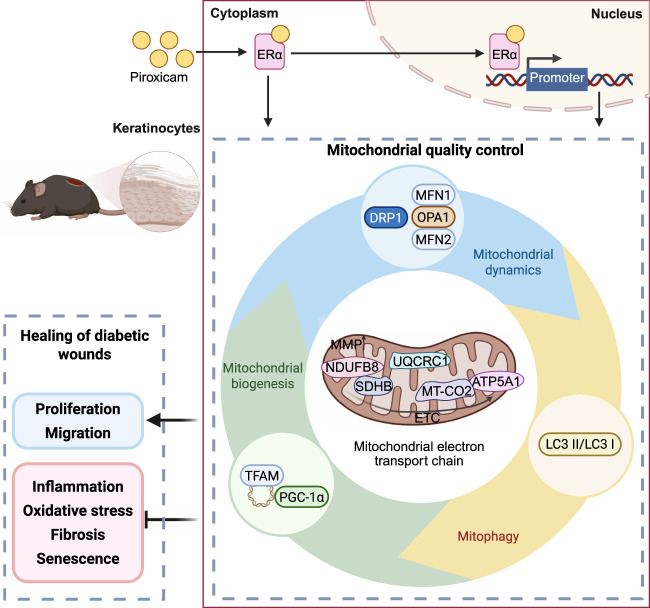

## Highlights

- Piroxicam, a traditional nonsteroidal anti-inflammatory drug, efficiently promotes the healing of diabetic foot ulcers.
- Piroxicam increases keratinocyte proliferation and migration and inhibits their senescence.
- Piroxicam exerts its pro-healing effects through restoring mitochondrial homeostasis by binding to ERα.
- Piroxicam’s pro-healing effects are closely related to its doses, which are vital in clinical use.

## 1 Introduction

Diabetic foot ulcer (DFU) represents one of the most common and severe chronic complications of diabetes, which is characterized by high incidence, high recurrence, and high rates of disability and mortality (Armstrong et al., 2017; Armstrong et al., 2023). By 2024, approximately 589 million adults worldwide and 148 million adults in China have diabetes. The large diabetic population has led to a continuous rise in the incidence of DFU (Genitsaridi et al., 2026). As revealed by a meta-analysis, among 5252 patients with DFU, 1861 (35.4%) experienced recurrence during follow-up (Lin et al., 2025). According to a prospective study, the recurrence rates of DFU at 1, 3, and 5 years were 42%, 58%, and 65%, respectively (Armstrong et al., 2025). The long-term prognosis of DFU remains poor, with an extremely high risk of amputation and mortality. The 5-year mortality rate stands at 49.1%, rising to 54%–79% following amputation (McDermott et al., 2023).

The treatment of DFU remains a significant clinical challenge. A network meta-analysis reported that after 12 weeks of standard treatments for DFU, including glycemic control, dressing, offloading, vascular assessment, and infection management, only 24% of patients achieved complete healing, highlighting the limited efficacy of conventional therapies (OuYang et al., 2024). Although negative-pressure wound therapy shows promise for DFU, its application remains constrained by several factors, including stringent patient selection criteria, high demands on equipment and procedural expertise, suboptimal patient adherence, and potential adverse effects on quality of life (Rys et al., 2020). Despite claims regarding the efficacy of platelet-rich plasma in DFU, its clinical utility is limited by inconsistent clinical outcomes, significant variability in therapeutic efficacy due to varying growth factor concentrations, and substantial inter-patient differences that may influence treatment response (Smith and Rai, 2024). Therefore, the severe clinical challenges underscore the pressing need to develop novel, targeted therapies and to discover new pharmacological targets for DFU.

Unlike acute wounds, DFU is characterized by non-resolving inflammation. In this setting, chronic hyperglycemia and immune cell dysfunction result in sustained M1 macrophage infiltration and the continuous release of cytokines such as IL-1β, IL-6, and TNF-α (Burgess et al., 2021). The non-resolving inflammation constitutes a self-amplifying vicious cycle that sustains a pathological epithelial-mesenchymal transition (EMT) state characterized by aberrant N-cadherin upregulation and persistent E-cadherin loss, thereby disrupting keratinocyte function and ultimately leading to prolonged wound non-healing (Youssef and Nieto, 2024). Hyperglycemia-induced non-resolving inflammation is mechanistically linked to mitochondrial dysfunction, in which elevated glucose boosts overproduction of reactive oxygen species (ROS) and oxidative stress, leading to the release of mitochondrial stress signals that sustain chronic inflammatory activation (Martinus and Goldsbury, 2018).

As a potent, long-acting non-steroidal anti-inflammatory drug and non-selective cyclooxygenase-2 (COX-2) inhibitor, piroxicam is particularly suitable for the oral and topical treatment of chronic inflammatory conditions such as arthritis (Brogden et al., 1981). However, whether piroxicam has therapeutic effects on non-resolving inflammation and the consequent impaired tissue repair in DFU remains largely unexplored. Given the availability of various approved transdermal formulations, repurposing piroxicam for DFU management holds significant translational potential and could be rapidly advanced into clinical application. Additionally, piroxicam is a traditional COX-2 inhibitor; emerging evidence suggests that it also exerts COX-independent effects by directly modulating mitochondrial function, acting as an uncoupler of oxidative phosphorylation (Moreno-Sanchez et al., 1999). If piroxicam proves beneficial for DFU healing, it raises the critical question of what therapeutic targets beyond COX-2 might mediate its effects. Given the male dominance in major and minor lower extremity amputations among diabetic patients and the beneficial effects of estrogen on wound healing and neovascularization in diabetic mice, we speculated that estrogen receptors may play pivotal roles and serve as potential pharmacological targets for DFU (Zhuge et al., 2018; Lopez-de-Andres et al., 2022). Investigating COX-2-independent mechanisms is of great significance, as it may uncover novel pharmacological targets and provide a rational basis for the development of new therapeutic strategies for DFU. Furthermore, piroxicam has been shown to exert protective effects against mitochondrial dysfunction and oxidative stress, as demonstrated by its ability to rescue neuronal cells from MPP + -induced toxicity by enhancing anaerobic ATP production and preserving cell viability (Soliman et al., 2009). Piroxicam has also been reported to provoke mitochondrial dysfunction and ROS overproduction, leading to oxidative injury, as evidenced by increased lipid peroxidation, antioxidant depletion, and caspase-3 activation (Abdeen et al., 2020). Given the dual-edge effects of piroxicam on mitochondrial function and oxidative stress, we hypothesized that piroxicam’s protective effects may be closely related to tissue and dose, which should be critically considered when exploring its clinical application strategies.

This study aimed to explore the beneficial effects of piroxicam on DFU, elucidate the molecular mechanisms by which piroxicam modulates mitochondrial function, and examine the influence of its dose. Using male and ovariectomized female C57BL/6 J diabetic mice and high-glucose-induced HaCaT cells, this study showed that piroxicam significantly promoted the healing of DFU *in vitro* and *in vivo* by alleviating mitochondrial dysfunction via binding to estrogen receptor alpha (ERα). This study highlights piroxicam’s significant potential for clinical use in DFU and proposes ERα as a novel pharmacological target.

## 2 Materials and methods

### 2.1 Establishment of C57BL/6 J DFU mouse models

Animal experiments strictly adhered to the National Research Council’s Guidelines for the Care and Use of Laboratory Animals, and the study protocol was approved by the Medical Ethics Committee of Sichuan University, China (Approval No.: K2020047). The 6-week-old male and 4-week-old female C57BL/6J mice were commercially obtained (Huafukang Biotechnology, Beijing, China). Mice were acclimated for 3 days under specific pathogen-free conditions with free access to food and water, housed in individually ventilated cages under controlled temperature and humidity, and maintained on a 12-h light/dark cycle.

**FIGURE 1:**
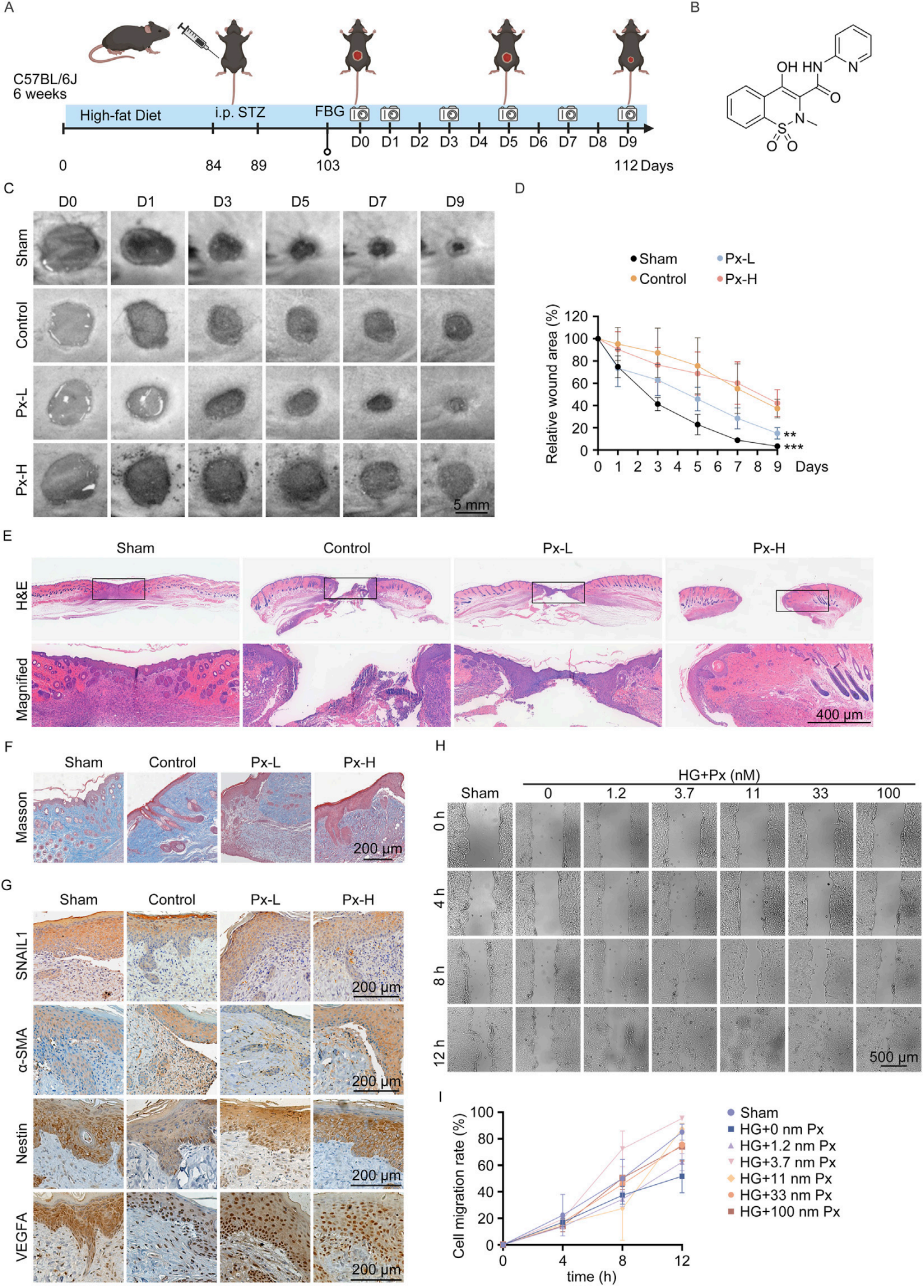
Piroxicam promoted wound healing in diabetic mice and enhanced the migration of high-glucose-induced HaCaT cells. **(A)** Male 6-week-old C57BL/6J mice were fed with an HFD for 84 days and subsequently intraperitoneally injected with STZ (50 mg/kg) for 5 consecutive days. Fourteen days after the last injection, mice with FBG above 11.3 mmol/L were included. A full-thickness skin excision was performed on the dorsal region to create a 6-mm diameter round wound. Piroxicam (1%, 0.33%) solution or saline was applied to the Px-H, Px-L, Control, and Sham groups, respectively, for 9 consecutive days. **(B)** The chemical structure of piroxicam. **(C)** The photographs of mice on days 0, 1, 3, 5, 7, and 9 post-puncture (scale bar = 5 mm). **(D)** Wound area was quantified using ImageJ software, and the relative wound area was calculated using the following formula: W_t_ = (A_0_ - A_t_)/A_0_ × 100%, where W_t_ was the relative wound area percentage at time t, A_0_ represented the initial wound area, and A_t_ represented the unhealed wound area on corresponding days (*n* = 6). **(E)** Wound epithelia closure of mice on day 9 post-puncture was assessed using H&E staining (scale bar = 400 μm). **(F)** Fibrosis of wounds on day 9 post-puncture was assessed using Masson’s trichrome staining (scale bar = 200 μm). **(G)** The expression of SNAIL1, α-SMA, Nestin, and VEGFA was determined with immunohistochemistry (scale bar = 200 μm). **(H)** HaCaT cells were induced with HG (60 mM) for 24 h, followed by treatment with piroxicam (0, 1.2, 3.7, 11, 33, and 100 nM). Cell migration capacity was assessed using a scratch assay (scale bar = 200 μm). **(I)** Cell migration rate (M_t_) of scratch images was performed as follows: M_t_ = (S_0_ - S_t_)/S_0_ × 100%, where S_0_ represented the scratch area at 0 h, and S_t_ denoted the scratch area at the corresponding time point. All data were presented as the mean ± SD. **P < 0.01, ***P < 0.001 compared to the Control group (*n* = 3). Abbreviations: HFD, high-fat diet; STZ, streptozotocin; FBG, fasting blood glucose; H&E, hematoxylin and eosin; SNAIL1, Snail family transcriptional repressor 1; α-SMA, α-smooth muscle actin; Nestin, neuroepithelial stem cell protein; VEGFA, vascular endothelial growth factor A; HG, high glucose; SD, standard deviation.

As shown in Figure 1A, male C57BL/6J mice were assigned to a sham group fed with standard chow or a model group fed with a high-fat diet (HFD, 60% fat energy ratio) for 84 days. Subsequently, the model group was intraperitoneally administered streptozotocin (STZ, Rhawn, Shanghai, China) at 50 mg/kg dissolved in sodium citrate buffer for 5 consecutive days, while the Sham group (n = 6) received an equal volume of sodium citrate buffer. Fourteen days after the last injection, fasting blood glucose (FBG) was measured by tail-vein blood sampling with a blood glucose meter (Sinocare, Changsha, China). Mice in the model group with FBG ≥ 11.3 mmol/L were considered diabetic and were further randomly divided into three groups of 6 mice each: the Control group, the Piroxicam low-dose (Px-L) group, and the Piroxicam high-dose (Px-H) group. Under inhalation anesthesia with isoflurane (RWD, Shenzhen, China), a 6-mm-diameter full-thickness circular wound was created on the mid-dorsum of mice using a sterile biopsy punch, with the day of excision designated as Day 0 (Zhou et al., 2025). Following initial photography, topical treatments were administered daily for nine consecutive days. Mice of the Sham and Control groups received saline. Piroxicam liniment (Figure 1B, PubChem CID: 54676228; CAS: 36322-90-4) with 1% piroxicam was purchased from Guangdong Hengjian Pharmaceutical Co., Ltd (Guangdong, China). The formulation of piroxicam liniment contains ethanol, laurocapram (Azone), diethanolamine, menthol, and propylene glycol as excipients. Mice in the Px-H group received the original piroxicam liniment, whereas mice in the Px-L group received a 1:3 dilution of the liniment with normal saline. No visible precipitation, crystallization, or phase separation was observed after dilution, and the preparation remained homogeneous throughout the experiment. Wound healing progress was recorded photographically every other day (Days 1, 3, 5, 7, and 9). Wound area was quantified using ImageJ (National Institutes of Health (NIH), Bethesda, MD, United States), and the relative wound area was calculated using the following formula: W_t_ = (A_0_ - A_t_)/A_0_ × 100%, where W_t_ was the relative wound area percentage at time t. A_0_ represented the initial wound area on day 0, and A_t_ represented the unhealed wound area on days 0, 1, 3, 5, 7, and 9. On Day 9, dorsal skin samples with a diameter of approximately 1 cm centered on the wound were harvested and fixed in 4% paraformaldehyde (PFA, Aladdin, Shanghai, China) overnight for subsequent paraffin embedding.

Female C57BL/6J mice were ovariectomized (OVX) under inhalation isoflurane-induced anesthesia (RWD, Shenzhen, China), followed by a two-week recovery period with a standard chow diet to ensure complete estrogen clearance. Then, female mice were subjected to identical modeling, grouping, intervention, and sampling procedures as male mice.

### 2.2 Cell culture and intervention

Human immortalized keratinocytes (HaCaT) and human embryonic kidney 293T (HEK293T) cells were both purchased from Haixing Biosciences (Suzhou, China). Cells were cultured in a minimum essential medium (MEM, BasalMedia, Shanghai, China) supplemented with 5.5 mM glucose, 10% fetal bovine serum (FBS), and 1% penicillin/streptomycin (P/S) at 37 °C in a 5% CO_2_ incubator. HaCaT cells cultured in standard complete MEM medium with 5.5 mM glucose served as the Sham group. HaCaT cells cultured in the MEM with high glucose (HG, 60 mM) for 24 h were used as the HG group. HG-induced HaCaT cells were further treated with piroxicam (1.2, 3.7, 11, 33, and 100 nM) for 24 h. A selective COX-2 inhibitor, celecoxib (1 μM, Aladdin, Shanghai, China), was used to determine the roles of COX-2. Tamoxifen (TAM, 15 μM, Solarbio, Beijing, China) was used to block ERα in HG-stimulated HaCaT cells, as reported in our previous study (Zou et al., 2022). A control group treated with HG plus tamoxifen alone was included in all relevant experiments to account for potential off-target effects.

### 2.3 Histological analysis

Skin tissues immersed overnight in 4% PFA were dehydrated and fixed, then embedded in paraffin. The paraffin-embedded tissues were sectioned into 8-μm-thick slices and stained with hematoxylin and eosin (H&E) to provide visual evidence for morphological analysis (Wick, 2019). Masson’s trichrome staining was used to assess collagen fibers and to evaluate tissue fibrosis. All images of paraffin-embedded tissue sections were acquired using a light microscope (Carl Zeiss, Jena, Germany).

### 2.4 Immunohistochemical staining assay

Paraffin-embedded skin tissue sections were dewaxed and hydrated. Immunohistochemical staining was performed as previously reported with slight modifications (Ladoire et al., 2012). After antigen retrieval with citrate buffer, sections were blocked with 2% bovine serum albumin (BSA) for 2 h. Tissue sections were incubated with Snail family transcriptional repressor 1 (SNAIL1, Proteintech Group, Wuhan, China, 13099-1-AP), α-smooth muscle actin (α-SMA, Sangon Biotech, Shanghai, China, D221592), Neuroepithelial stem cell protein (Nestin, Proteintech Group, 19483-1-AP), vascular endothelial growth factor A (VEGFA, Proteintech Group, 19003-1-AP), caveolin-1 (CAV-1, Sangon Biotech, D161423), interleukin-1 beta (IL-1β, Proteintech Group, 16806-1-AP), and interleukin-18 (IL-18, Proteintech Group, 10663-1-AP) at 4 °C overnight, followed by incubation with secondary antibodies (Invitrogen, Carlsbad, CA, United States of America) at room temperature for 1 h. Stains were developed using a 3,3′-diaminobenzidine (DAB) solution (Beijing Zsgb Bio, Beijing, China). Sections were subsequently counterstained with hematoxylin and mounted with neutral resin. Images were taken using a light microscope (Carl Zeiss).

### 2.5 Cell scratch assay

Upon reaching full confluence, a cross-shaped scratch was made at the center of each well using a 200 μL pipette tip. Cell debris was washed away with phosphate-buffered saline (PBS). Images were captured at the same scratch location under a light microscope (Olympus, Tokyo, Japan) at 20× magnification at 0, 4, 8, and 12 h post-scratch. Analysis of scratch images was performed using ImageJ (NIH) to calculate migration rate (M_t_) by the following formula: M_t_ = (S_0_ - S_t_)/S_0_ × 100%, where S_0_ represented the scratch area at 0 h, and S_t_ denoted the scratch area at the corresponding time point.

### 2.6 EdU assay

HaCaT cell proliferation was assessed using the BeyoClick^TM^ EdU-647 proliferation kit (Beyotime Biotechnology, Shanghai, China) following the manufacturer’s instructions. EdU staining solution was added to HaCaT cells, which were incubated for 2 h at 37 °C in a 5% CO_2_ incubator. After being washed with PBS, cells were fixed with 4% PFA for 20 min, permeabilized with 0.5% Triton X-100 for 15 min, and incubated in the dark with the click reaction solution for 30 min. Nuclei were stained with Hoechst 33258 (Sigma-Aldrich, St. Louis, MO, United States of America) for 5 min. Images were acquired using an inverted fluorescence microscope (Carl Zeiss, Jena, Germany). Cell proliferation rates were analyzed using ImageJ (NIH).

### 2.7 Senescence-associated β-galactosidase (SA-β-gal) staining assay

HG-induced HaCaT cells were treated with piroxicam for 24 h, and washed twice with PBS. Then, cells were stained with the SA-β-gal staining kit (Beyotime Biotechnology, Shanghai, China) according to the manufacturer’s instructions. Cells were fixed with 4% PFA solution for 15 min. After being washed three times with PBS, cells were stained with the SA-β-gal staining solution overnight at 37 °C in the dark, without CO_2_. Senescent cells were identified by blue-green staining. Images were taken with a light microscope (Carl Zeiss).

### 2.8 Dihydroethidium (DHE) fluorescence staining assay

After being incubated with piroxicam, HaCaT cells were washed twice with PBS. Then, the cells were stained with a DHE probe (20 μM, Aladdin, Shanghai, China) at 37 °C for 30 min in darkness. Intracellular DHE fluorescence was visualized and captured using an inverted fluorescence microscope (Carl Zeiss) and analyzed using ImageJ (NIH).

### 2.9 MitoSOX fluorescent staining assay

Mitochondrial ROS (mtROS) were detected using the MitoSOX fluorescent staining assay. Cells were stained with MitoSOX (Thermo Fisher, Waltham, MA, United States) solution (10 μM) at 37 °C for 30 min in the dark. After being rinsed twice with a pre-heated serum-free medium, images were taken using an inverted fluorescence microscope (Carl Zeiss) and analyzed using ImageJ (NIH).

### 2.10 Mitochondrial membrane potential (MMP) determination assay

MMP was detected using a 5,5′,6,6′-tetrachloro-1,1′,3,3′-tetraethylbenzimidazolylcarbocyanine iodide (JC-1) staining probe (MedChemExpress, Monmouth Junction, NJ, United States) following the manufacturer’s instructions. Cells were incubated with the JC-1 working solution (2.5 μM) at 37 °C for 30 min. Images were then captured using an inverted fluorescence microscope (Carl Zeiss) and analyzed using ImageJ (NIH).

Tetramethylrhodamine methyl ester (TMRM) staining was also employed to detect MMP. HaCaT cells were stained with a TMRM solution (150 nM, Sangon Biotech) at 37 °C for 30 min. Images were then captured using an inverted fluorescence microscope (Carl Zeiss) and analyzed in ImageJ (NIH).

### 2.11 Immunofluorescence assay

Immunofluorescence staining was performed as previously reported with slight modifications (Xie et al., 2024). Cells were fixed with 4% PFA for 20 min, permeabilized with 0.5% Triton X-100, and blocked with 4% BSA for 1 h at room temperature. Then cells were incubated with primary antibodies against histone H2AX phosphorylated at Ser139 (γ-H2AX, Beyotime Biotechnology, Shanghai, China, AF5836), ataxia telangiectasia mutated phosphorylated at Ser1981 (p-ATM, Proteintech Group, Wuhan, China, 39529), translocase of outer mitochondrial membrane 20 (Tom20, Proteintech Group, 11802-1-AP), and galactosidase beta 1 (GLB1, Sangon Biotech, D290580) overnight at 4 °C in the darkness. After being washed three times with PBS, cells were incubated with secondary antibodies (Jackson Immuno, West Grove, PA, United States) in the dark. Finally, nuclei were stained with Hoechst 33258 (Sigma-Aldrich, St. Louis, MO, United States) for 5 min. Images were acquired using a confocal microscope (Carl Zeiss). The acquired images were imported into ZEN software for processing and analyzed using ImageJ (NIH).

### 2.12 Construction of stable HaCaT cell lines and multicolor fluorescence imaging

Lentivirus (Lv) encoding pLenti-CMV-mCherry-GFP-LC3-IRES-Puro-WPRE, pLenti-CMV-MitoTimer-Puro-WPRE, and pLenti-CMV-mt-Keima-Puro-WPRE were commercially obtained from OBIO Tech (Shanghai, China). HaCaT cells were passaged at a 1:10 dilution and cultured at 37 °C in a 5% CO_2_ environment for 24 h. Then, the cells were infected with Lv for 48 h. Stable cell lines were selected by being incubated with puromycin (0.06, 0.18, 0.55, 1.67, and 5.00 μg/mL) for 24 h. Cells exhibiting good condition with a high positive rate were transferred to complete medium for further culture and stored at −80 °C for subsequent experiments.

HaCaT cells stably expressing mCherry-GFP-LC3 were fixed with 4% PFA for 20 min, permeabilized with 0.5% Triton X-100 (Sangon Biotech) for 15 min, and blocked with 4% BSA for 1 h. For autophagic flux assessment, nuclei were stained with Hoechst 33258 (Sigma-Aldrich) for 5 min. For mitophagy assessment, cells were incubated with an anti-Tom20 antibody (Proteintech Group, 11802-1-AP), followed by a secondary antibody, and the colocalization of LC3 puncta and Tom20 signals was analyzed using ImageJ. All images were acquired using a confocal microscope (Carl Zeiss).

HaCaT cells stably expressing mt-Keima were used to detect mitophagy. HaCaT cells stably expressing MitoTimer were used to measure mitochondrial turnover. Cells were fixed, permeabilized, blocked, and nuclei were stained using the same protocol as for HaCaT cells stably expressing mCherry-GFP-LC3. Images were then acquired using a confocal microscope (Carl Zeiss).

### 2.13 MitoTracker staining assay

After being washed twice with pre-warmed PBS, HaCaT cells were stained with MitoTracker Red CMXRos (Thermo Fisher, Waltham, MA, United States) at 37 °C for 15 min in the dark. Images were acquired using a confocal microscope (Carl Zeiss).

### 2.14 Cytoskeleton-mitochondria co-staining assay

HaCaT cells were stained with the primary antibody against Tom20 (Proteintech Group, Wuhan, China, 11802-1-AP) overnight at 4 °C in the dark. After being washed three times with PBS, cells were incubated with secondary antibodies (Jackson Immuno, West Grove, PA, United States) in the dark. Then, the cells were further stained with a phalloidin solution (Thermo Fisher, Waltham, MA, United States) at 100 nM for 30 min in the dark. Cells were washed with PBS, and nuclei were stained with Hoechst 33258 (Sigma-Aldrich, St. Louis, MO, United States) for 5 min. Images were acquired using a confocal microscope (Carl Zeiss).

### 2.15 Molecular docking

The crystal structure of the ligand-binding domain (LBD) of ERα was downloaded from the RCSB Protein Data Bank database. A high-resolution crystal structure of the LBD (PDB Code: 1A52, resolution: 2.00 Å) was retrieved and selected as the receptor for molecular docking. Protein structures were standardized using the Protein Preparation Wizard in the Schrödinger Maestro platform (Schrödinger, LLC, Cambridge, United States). This process included removing co-crystallized ligands and crystallization water molecules, adding hydrogen atoms, and optimizing the hydrogen bond network to obtain a stable receptor conformation. The three-dimensional structural data for piroxicam (PubChem CID: 54676228, CAS No.: 36322-90-4) were obtained from the PubChem database (https://pubchem.ncbi.nlm.nih.gov/). After downloading, the data were processed and converted to a MOL2 file, serving as the ligand input structure for molecular docking. The binding site was determined based on the known pocket region of the ERα ligand-binding domain. Molecular docking was performed using the Glide module, with the receptor held rigid and the ligand undergoing a flexible conformational search, while other parameters were set to default values. Obtained conformations were ranked according to GlideScore, and the conformation with the highest score and a reasonable binding mode was selected for subsequent analysis. The alignment results were visualized in Maestro and PyMOL 2.4 (Schrödinger, LLC, Cambridge, United States), with distance measurements and structural analysis of hydrogen bonds and aromatic interactions between key amino acid residues.

### 2.16 Cellular thermal shift assay (CETSA)

CETSA was performed as previously reported (Zhou et al., 2025). When 293 T cells reached 80% confluence, the medium was replaced with either piroxicam (33 nM) or E2 (10 nM) at 37 °C for 8 h. Then, the cells were placed on ice, and cell lysis buffer supplemented with phenylmethylsulfonyl fluoride (PMSF, Yuanye, Shanghai, China) was added. The cell suspensions from each group were divided into eight aliquots and transferred to PCR tubes for gradient heating from 37 °C to 62 °C for 4 min in a T100 thermal cycler (Bio-Rad, Hercules, CA, United States). After heating, the suspensions underwent three freeze-thaw cycles between liquid nitrogen and a 37 °C water bath to ensure complete lysis. Then the suspensions were centrifuged at 20000×g for 20 min at 4 °C. The supernatant was transferred to new 1.5 mL centrifuge tubes, mixed with 5× sodium dodecyl sulfate-polyacrylamide gel electrophoresis (SDS-PAGE) sample loading buffer, and incubated at 98 °C for 10 min. Finally, the levels of the target proteins were detected using Western blotting.

### 2.17 Nuclear-cytoplasmic segregation experiment

The nuclear-cytoplasmic separation was performed with slight modifications as previously described (Senichkin et al., 2021). HaCaT cells were digested with trypsin and collected into 1.5 mL tubes. After washing with ice-cold PBS, cells were resuspended in a hypotonic buffer containing 0.1% NP-40 and mechanically disrupted with a tissue grinder with zirconia beads on ice. The homogenate was centrifuged at 2000×g for 20 min at 4 °C. The supernatant was subjected to a second centrifugation at 15,000×g for 20 min at 4 °C. The obtained supernatant, representing the cytoplasmic fraction, was mixed with 87.5 μL of 5× SDS-PAGE sample loading buffer and boiled at 98 °C for 10 min. The precipitate obtained from the first centrifugation was washed with isotonic buffer containing 0.1% NP-40 and centrifuged again at 2000×g for 10 min at 4 °C. The resulting pellet was resuspended in cell lysis buffer, incubated on ice for 20 min, and centrifuged again at 2000×g for 20 min at 4 °C. The supernatant representing the nuclear soluble fraction was then mixed with 5× SDS-PAGE sample loading buffer and boiled at 98 °C for 10 min. Subsequently, all samples were analyzed by Western blotting to detect the levels of the target proteins.

### 2.18 Western blotting assay

Western blotting was conducted as previously reported with certain adjustments (Zhang et al., 2017). Briefly, cells were lysed using 5× SDS-PAGE sample loading buffer, boiled at 98 °C for 10 min, and centrifuged at 13680×g for 3 min at room temperature. Equal amounts of the supernatant were subjected to SDS-PAGE and transferred to polyvinylidene fluoride (PVDF) membranes. After being blocked with 5% fat-free milk in Tris-buffered saline containing 0.1% Tween 20 at room temperature for 2 h, the PVDF membranes were incubated with primary antibodies against epithelial cadherin (E-cadherin, Proteintech Group, Wuhan, China, 20874-1-AP), neuronal cadherin (N-cadherin, Proteintech Group, 22018-1-AP), SNAIL1 (Proteintech Group, 13099-1-AP), collagen type I (Collagen-I, Sangon Biotech, Shanghai, China, D290319), β-tubulin (Biodragon, Suzhou, China, B1031), α-SMA (Sangon Biotech,D221592), transforming growth factor-β (TGF-β, Proteintech Group, 21898-1-AP), SMAD family member 2 (SMAD2, Sangon Biotech, D164586), matrix metallopeptidase 9 (MMP9, Sangon, D120097), glyceraldehyde-3-phosphate dehydrogenase (GAPDH, Proteintech Group,10494-1-AP), β-galactosidase (GLB1, Sangon Biotech, D290580), tumor protein p53 (P53, Sangon Biotech, D199442), CAV-1 (Sangon Biotech, D161423), BCL2-associated X protein (Bax, Sangon Biotech, D220073), B-cell lymphoma-2 (Bcl-2, Sangon Biotech, D290158), IL-1β (Proteintech Group, 10806-1-AP), IL-18 (Proteintech Group, 10663-1-AP), nuclear factor kappa B (NF-κB, Proteintech Group, 14220-1-AP), phospho-NF-κB p65 (Ser536) (p-RELA, Cell Signaling Technology, Danvers, MA, United States of America, 3033S), phospho-NF-κB2 p100 (Ser866) (p-NFKB2, Cell Signaling Technology, 4810S), cyclic GMP-AMP synthase (cGAS, Sangon Biotech, D163570), stimulator of interferon genes (STING, Proteintech Group, 19851-1-AP), NOD-like receptor family pyrin domain containing 3 (NLRP3, Proteintech Group,27458-1-AP), superoxide dismutase 1 (SOD1, Proteintech Group,10269-1-AP), superoxide dismutase 2 (SOD2, Proteintech Group, 24127-1-AP), NAD(P)H quinone dehydrogenase 1(NQO1, Sangon Biotech, D261049), nuclear respiratory factor 1 (NRF1, Proteintech Group, 66832-1-Ig), nuclear factor erythroid 2-related factor 2 (NRF2, Proteintech Group, 16396-1-AP), mitofusin 1 (MFN1, Proteintech Group, 13798-1-AP), mitofusin 2 (MFN2, Proteintech Group, 12186-1-AP), optic atrophy 1 (OPA1, ABclonal, Wuhan, China, A9833), peroxisome proliferator-activated receptor gamma coactivator 1-alpha (PGC-1α, Proteintech Group, 66369-1-Ig), mitochondrial transcription factor A (TFAM, Proteintech Group, 22586-1-AP), heat shock protein 60 (HSP60, Sangon Biotech, D194953), translocase of inner mitochondrial membrane 23 (Tim23, BD, Franklin Lakes, NJ, United States, 611223), Tom20 (Proteintech Group, 11802-1-AP), dynamin-related protein 1 (DRP1, Sangon Biotech, D198957), microtubule-associated protein 1A/1B-light chain 3 (LC3, MBL, Nagoya, Aichi, Japan, M1863), sequestosome 1 (SQSTM1, Proteintech Group, 18420-1-AP), Beclin1 (Sangon Biotech, D160120), oxidative phosphorylation (OXPHOS, Proteintech Group, PK30006), estrogen receptor alpha (ERα, Proteintech Group, 20698-1-AP), estrogen receptor beta (ERβ, Proteintech Group, 14007-1-AP), histone H3 (Proteintech Group, 17168-1-AP), and β-actin (Santa Cruz Biotechnology, Dallas, TX, United States, SC-47778). After incubation with horseradish peroxidase-conjugated secondary antibodies (Invitrogen, Carlsbad, CA, United States), enhanced chemiluminescence (Biodragon) was applied to visualize the bands using a chemiluminescence imager (Clinx, Shanghai, China). The densitometric analysis was performed using ImageJ (NIH).

### 2.19 Statistical analysis

All data were expressed as mean ± standard deviation (SD), with each experiment independently replicated three times. Statistical analysis was performed using GraphPad Prism 8.0 software (GraphPad software Inc., La Jolla, CA, United States). Statistical differences were analyzed using one-way analysis of variance (ANOVA) and two-way ANOVA, and *P* < 0.05 was considered statistically significant.

## 3 Results

### 3.1 Piroxicam promoted DFU healing *in vivo* and *in vitro*

To investigate the effects of piroxicam on wound healing of DFU, male C57BL/6J mice were employed to establish a DFU model. As shown in Figures 1C,D, mice in the Control group exhibited a significantly delayed healing process compared with those in the Sham group throughout the entire healing period (*P* < 0.05). The peak difference occurred on Day 5, with the relative wound area in the Control group 53% higher than in the Sham group. Low-dose piroxicam (0.33%) showed significant pro-healing effects. The relative wound area of the Px-L group was 30% on day 5, 27% on day 7, and 22% on day 9, while the wound area of the Control group was 53% on day 5, 46% on day 7, and 34% on day 9 (*P* < 0.05). Notably, there was no statistical difference between the wound healing of the Px-L group and that of the Sham group at any time point, indicating that low-dose piroxicam restored the tissue repair of diabetic mice to a level comparable to that in healthy mice. Surprisingly, the efficacy of high-dose piroxicam (1%) was significantly weaker than that of the low-dose piroxicam. There was no statistical difference in the relative wound area between the Px-H group and the Control group, suggesting that piroxicam does not promote DFU healing at high dose. Histological analysis of wound epithelium was consistent with the wound closure outcomes. Wounds in the Control group exhibited significantly increased epidermal thickness, more pronounced hyperkeratosis, and delayed re-epithelialization compared with those in the Sham group, indicating impaired wound healing. Low-dose piroxicam treatment resulted in a more intact epidermal structure, with significantly reduced epidermal thickness compared with the Control group, suggesting that piroxicam effectively promotes re-epithelialization and tissue repair of DFU at low dose (Figure 1E; Supplementary Figure S1). Masson’s trichrome staining was further used to evaluate collagen deposition and remodeling in the wound area (Figure 1F). The results showed that, in the Control group, collagen fibers were loosely arranged and disorganized, indicating impaired remodeling. After piroxicam treatment, collagen fibers in the wound area appeared denser, more orderly, and more aligned. Quantitative analysis of the blue collagen-positive area (Supplementary Figure S1) further demonstrated that collagen deposition was significantly increased in the low-dose piroxicam group compared with the Control group, suggesting that piroxicam promotes collagen remodeling and dermal maturation. Additionally, immunohistochemical staining was performed to detect SNAIL1 and α-SMA expression, thereby assessing epithelialization and fibrosis. As shown in Figure 1G, the intensity of SNAIL1-positive staining in basal layer cells of the Sham group was significantly higher than that in the control group. The reduced SNAIL1 expression in the Control group indicates impaired keratinocyte activation and wound healing. Piroxicam partially restored Snail1 expression. Furthermore, α-SMA positive signals of the Control group were significantly higher than those of the Sham group, while α-SMA positive staining was markedly reduced in the Px-L group. In contrast, α-SMA expression levels in the Px-H group remained comparable to those of the Control group. Additionally, the expression of key angiogenic molecules, Nestin and VEGFA, was determined by immunohistochemistry (Figure 1G). Compared with the Sham group, Nestin-positive staining was significantly reduced in the wound area of the Control group, suggesting that neovascularization was suppressed in chronic hyperglycemia. Following piroxicam treatment, Nestin expression levels increased significantly, with the Px-L group showing a more pronounced improvement. Consistent with this, VEGFA-positive expression in the Control group was significantly lower than that in the Sham group, whereas VEGFA expression was markedly restored by piroxicam, with the Px-L group showing superior recovery compared to the Px-H group. These results indicate that piroxicam promotes angiogenesis in DFU, thereby creating favorable conditions for wound healing.

To further investigate the impact of piroxicam on re-epithelialization in DFU, HG-induced HaCaT cells were used. As shown in Figures 1H,I, HG significantly decreased HaCaT cell migration (52% vs. 85%), an effect enhanced by piroxicam. The migration rate was 62%, 95%, 88%, 75%, and 74% when incubated with piroxicam at 1.2, 3.7, 11, 33, and 100 nM, respectively. The migration rate of all piroxicam-treated groups exceeded that of the Control group, with even the 3.7 nM and 11 nM groups surpassing the Sham group. Both *in vivo* and *in vitro* results supported that piroxicam exerts its best effects at low doses.

### 3.2 Piroxicam enhanced proliferation, reversed the non-canonical EMT program, and decreased fibrosis and cellular senescence in HG-induced HaCaT cells or male diabetic mice

**FIGURE 2:**
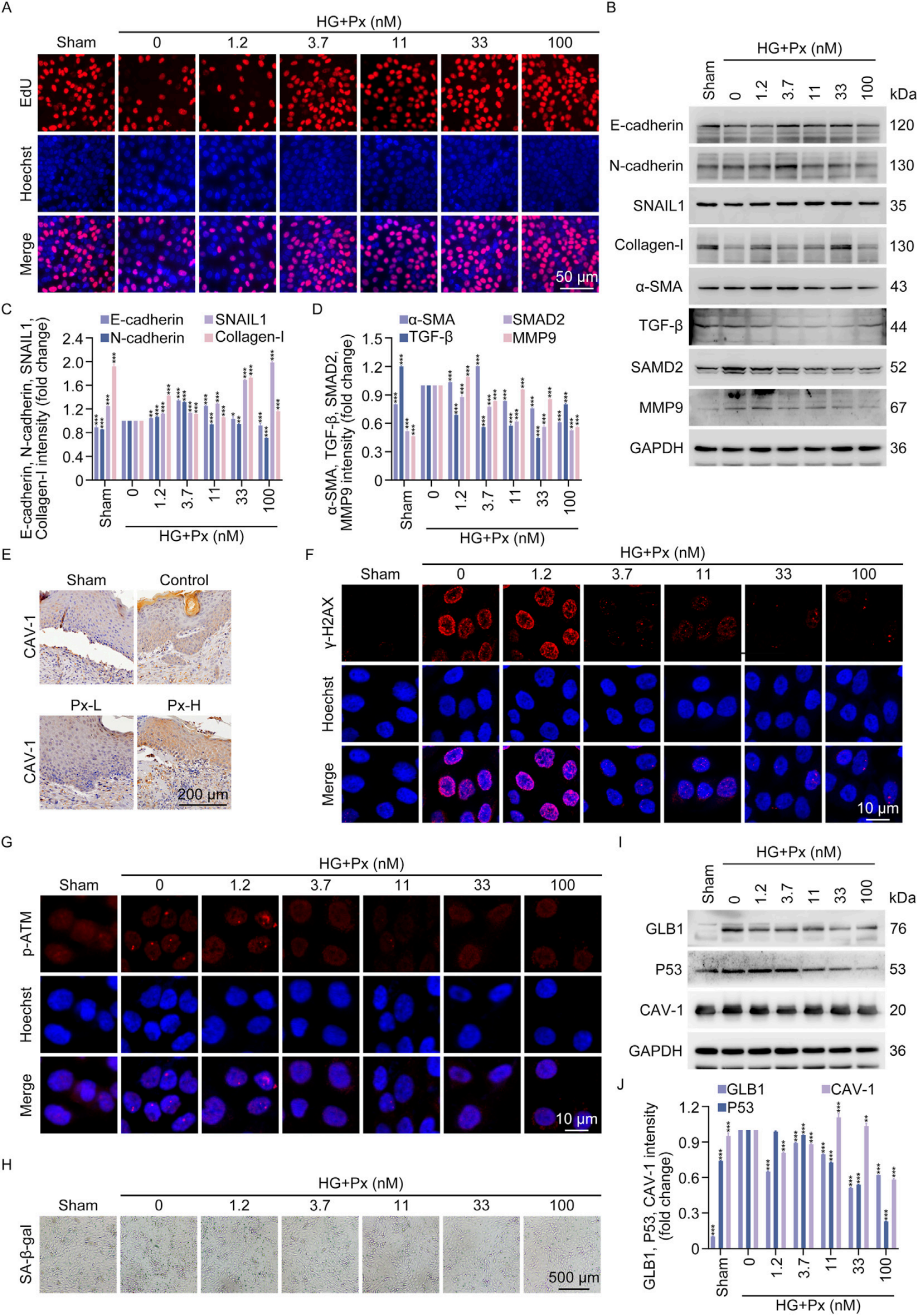
Piroxicam promoted proliferation, reversed the aberrant EMT phenotype, and inhibited fibrosis and cellular senescence in HG-induced HaCaT cells or in male diabetic mice. **(A)** HG-induced HaCaT cells were treated with piroxicam (0, 1.2, 3.7, 11, 33, 100 nM) for 24 h, and EdU staining was used to assess the proliferation (scale bar = 50 μm). **(B)** The expression levels of EMT-related proteins (E-cadherin, N-cadherin, SNAIL1, Collagen-I) and fibrosis-related proteins (α-SMA, TGF-β, SMAD2, MMP9) were detected by Western blotting. **(C)** Relative quantitative analysis of epithelial-mesenchymal transition-related proteins (E-cadherin, N-cadherin, SNAIL1, Collagen-I) was performed. **(D)** Relative quantitative analysis of fibrosis-related proteins (α-SMA, TGF-β, SMAD2, MMP9) was performed. **(E)** The expression of CAV-1 in the skin around wounds of male DFU mice was measured using immunohistochemical staining (scale bar = 200 μm). **(F)** Immunofluorescence staining was used to detect the expression of γ-H2AX in HG-induced HaCaT cells (scale bar = 10 μm). **(G)** Immunofluorescence staining was used to analyze the expression of p-ATM in HG-induced HaCaT cells (scale bar = 10 μm). **(H)** Representative images of HaCaT cells as stained by SA-β-gal (scale bar = 500 μm). **(I)** Western blotting was employed to determine the expression of senescence-related proteins, including GLB1, P53, and CAV-1. **(J)** Relative quantitative analysis of senescence-related proteins (GLB1, P53, CAV-1) was performed. All data were presented as mean ± SD. *P < 0.05, **P < 0.01, ***P < 0.001 compared to the 0 nM group (*n* = 3). Abbreviations: HG, high glucose; EMT, epithelial-mesenchymal transition; E-cadherin, epithelial cadherin; N-cadherin, neural cadherin; SNAIL1, Snail family transcriptional repressor 1; Collagen-I, Collagen type 1; α-SMA, α-smooth muscle actin; TGF-β, transforming growth factor-beta; SMAD2, small mothers against decapentaplegic 2; MMP9, matrix metalloproteinase-9; CAV-1, caveolin-1; γ-H2AX, phosphorylated histone H2AX (Ser139); p-ATM, phosphorylated ataxia-telangiectasia mutated; SA-β-gal, senescence-associated β-galactosidase; GLB1, galactosidase beta 1; SD, standard deviation.

The effects of piroxicam on the proliferation under HG were assessed using EdU fluorescence staining (Figure 2A). Results showed that HG significantly suppressed cellular DNA synthesis as evidenced by a substantial reduction in EdU-positive signals compared with the Sham group. Piroxicam increased the proliferation of HG-induced HaCaT cells in a marked dose-dependent manner. The effects of piroxicam became apparent at 3.7 nM and progressively intensified with increasing concentration, with proliferation levels approaching those of the Sham group between 11–100 nM (Supplementary Figure S1). Western blot analysis showed that HG significantly decreased the expression of the epithelial marker E-cadherin, the repair-associated transcription factor SNAIL1, and Collagen-I, while increasing the expression of the mesenchymal marker N-cadherin. Piroxicam treatment partially reversed these changes. These findings suggest that HG disrupted epithelial homeostasis and cell adhesion in HaCaT cells, accompanied by decreased collagen production, which was partially reversed by Piroxicam (Figures 2B,C). These changes may contribute to impaired wound healing in DFU, which was reversed by piroxicam as evidenced by restored E-cadherin expression, suppressed N-cadherin, and upregulated SNAIL1 and Collagen-I. These changes indicated that piroxicam induced a shift toward a more regulated epithelial phenotype and improved extracellular matrix deposition, which may facilitate wound healing of DFU. Furthermore, key fibrosis markers were determined by Western blotting. As shown in Figures 2B,D, HG activated the TGF-β/SMAD2 pathway, upregulating α-SMA and MMP9, indicating a profibrotic program. The suppression of piroxicam on these molecules suggested that piroxicam inhibited the TGF-β/SMAD2-mediated fibrogenic signaling, thereby attenuating the fibrotic program induced by HG.

Given that aberrant EMT and fibrosis often result from persistent cellular stress, we next investigated whether HG induced cellular senescence, a key driver of tissue dysfunction. As shown in Figure 2E, HG-induced upregulated CAV-1 expression in the basal layer of skin tissue was significantly decreased by low-dose piroxicam, but not high-dose. Consistently, the upregulation of γ-H2AX and p-ATM induced by HG indicated the activation of a DNA damage response, a well-established trigger of cellular senescence (Figures 2F,G; Supplementary Figure S1). The suppression of piroxicam on these showed that piroxicam protects cells from genotoxic stress, thereby potentially alleviating stress-induced premature senescence. Moreover, SA-β-gal staining results revealed that HG induced dense blue senescence-positive signals in HaCaT cells, which were significantly decreased by piroxicam (Figure 2H; Supplementary Figure S1). Additionally, HG significantly upregulated GLB1, a famous senescence-associated β-galactosidase, along with P53 and CAV-1, indicating that HG induced a senescent phenotype in keratinocytes. The suppression of these proteins by piroxicam suggested that it protects cells from stress-induced premature senescence (Figures 2I,J). Collectively, HG induced severe functional impairment and pathological damage in cells, while piroxicam increased cell proliferation, reversed aberrant EMT and fibrosis, and mitigated cellular senescence both *in vivo* and *in vitro*.

### 3.3 Piroxicam ameliorated HG-induced inflammation, oxidative stress, and apoptosis

**FIGURE 3:**
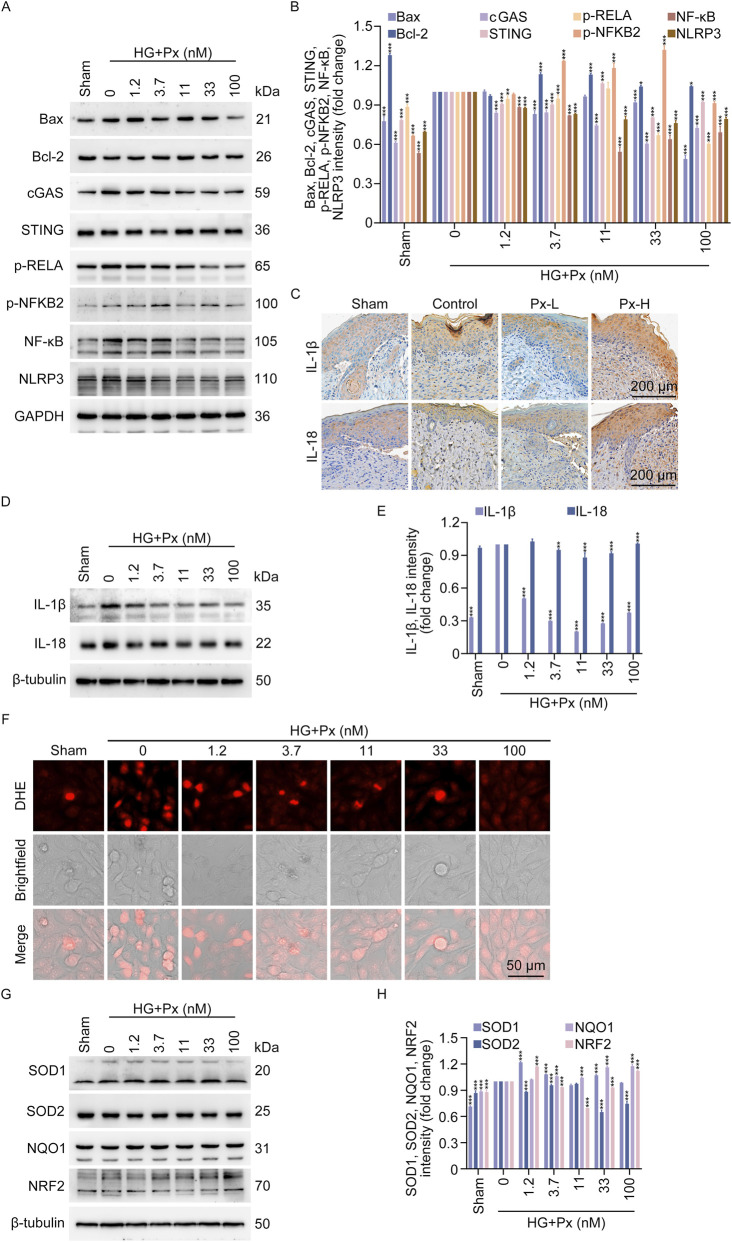
Piroxicam inhibited apoptosis, inflammation, and oxidative stress. **(A)** HG-induced HaCaT cells were treated with piroxicam (0, 1.2, 3.7, 11, 33, 100 nM) for 24 h. The expression levels of key molecules in apoptosis and inflammation were determined using Western blotting, including Bax, Bcl-2, NF-κB, and its phosphorylated forms at Ser536 (p-RELA) and Ser866 (p-NFKB2), cGAS, and NLRP3. **(B)** Quantitative analysis of Bax, Bcl-2, NF-κB, phospho-NF-κB p65 (Ser536, p-RELA), phospho-NF-κB2 p100 (Ser866, p-NFKB2), cGAS, STING, and NLRP3 protein levels was performed using ImageJ. **(C)** The expression of IL-1β and IL-18 in the skin tissue of male mice with DFU was immunohistochemically stained (scale bar = 200 μm). **(D)** The expression of IL-1β and IL-18 of HG-induced HaCaT cells was determined using Western blotting. **(E)** Quantitative analysis of IL-1β and IL-18 protein levels was performed using ImageJ. **(F)** HG-induced HaCaT cells were treated with piroxicam and then incubated with a DHE probe to detect intracellular ROS levels, which were observed under an inverted fluorescence microscope (scale bar = 50 μm). **(G)** The expression of key proteins in oxidative stress, including SOD1, SOD2, NRF2, and NQO1, was detected by Western blotting. **(H)** Quantitative analysis of protein levels for SOD1, SOD2, NQO1, and NRF2 was performed using ImageJ. All data were presented as mean ± SD. *P < 0.05, **P < 0.01, ***P < 0.001 compared to the 0 nM group (*n* = 3). Abbreviations: HG, high glucose; SD, standard deviation; Bcl-2, B-cell lymphoma 2; Bax, Bcl-2-associated X protein; NF-κB, nuclear factor κB; cGAS, cyclic GMP-AMP synthase; p-RELA, phosphorylated NF-κB (p65) at Ser536; p-NFKB2, phosphorylated NF-κB (p100) at Ser866; NLRP3, NOD-like receptor family pyrin domain containing 3; IL-1β, interleukin-1β; IL-18, interleukin-18; DHE, dihydroethidium; ROS, reactive oxygen species; SOD1, superoxide dismutase 1; SOD2, superoxide dismutase 2; NRF2, nuclear factor erythroid 2-related factor 2; NQO1, NAD(P)H:quinone oxidoreductase 1.

When HG-induced DNA damage exceeds the cellular repair capacity, persistent senescence may ultimately shift towards apoptosis to eliminate irreversibly damaged cells. The expression of anti-apoptotic protein Bcl-2 and the pro-apoptotic protein Bax in HG-induced HaCaT cells was determined. As shown in Figures 3A,B, HG significantly upregulated the Bax/Bcl-2 ratio, whereas piroxicam suppressed apoptotic signaling activation and attenuated apoptosis. Notably, DNA fragments released during apoptosis may be recognized by cGAS, which subsequently activated STING and its downstream NF-κB and NLRP3 inflammasome, thereby amplifying cell death signals into inflammatory responses. As shown in Figures 3A,B, Western blotting revealed that HG significantly increased cGAS and STING levels, which were reversed by piroxicam. Concurrently, HG significantly upregulated the phosphorylated forms of NF-κB, including p-RELA (phosphorylated NF-κB p65 at Ser536) and p-NFKB2 (phosphorylated NF-κB p100 at Ser866), reflecting a robust activation of inflammatory responses. Furthermore, the expression of the inflammasome component NLRP3 was also increased by HG. These changes were all abrogated by piroxicam, and the strongest effects occurred at low doses. The piroxicam’s effects on inflammation were also detected. As shown in Figure 3C, the epidermal basal layer of the Control group showed abundant expression of IL-1β and IL-18 compared to the Sham group. Piroxicam significantly inhibited the secretion of IL-1β and IL-18 at low doses, while piroxicam even increased their secretion at high doses. Consistently, the protein expression levels of IL-1β and IL-18 of HG-induced HaCaT cells were also significantly attenuated by piroxicam (Figures 3D,E).

Given that the inflammasome and NF-κB activation are often mediated by redox-sensitive signaling pathways, the intracellular ROS levels were further detected using DHE staining. As shown in Figure 3F; Supplementary Figure S1, HG boosted the red fluorescence intensity of DHE, indicating significant ROS accumulation, whereas piroxicam significantly reduced intracellular ROS. Expressions of key proteins in the antioxidant defense system were determined (Figures 3G,H). Piroxicam significantly upregulated NRF2, the core transcription factor of the antioxidant response, as well as its downstream antioxidant proteins NQO1 and SOD1, whereas SOD2 expression was decreased compared with the HG group. Combined with the reduced ROS levels, these findings suggest that piroxicam alleviated HG-induced oxidative stress by enhancing cellular antioxidant capacity. Notably, SOD1 and SOD2 were localized in the cytoplasm and mitochondria, respectively, and they coordinated to dynamically regulate antioxidant responses. Increased SOD2 expression under HG may reflect compensatory responses induced by sustained mtROS accumulation, whereas piroxicam decreased SOD2 expression, indicating relief of oxidative stress and reduced compensatory demand.

### 3.4 Piroxicam alleviated HG-induced mitochondrial oxidative stress, promoted mitophagy, and restored mitochondrial function

**FIGURE 4:**
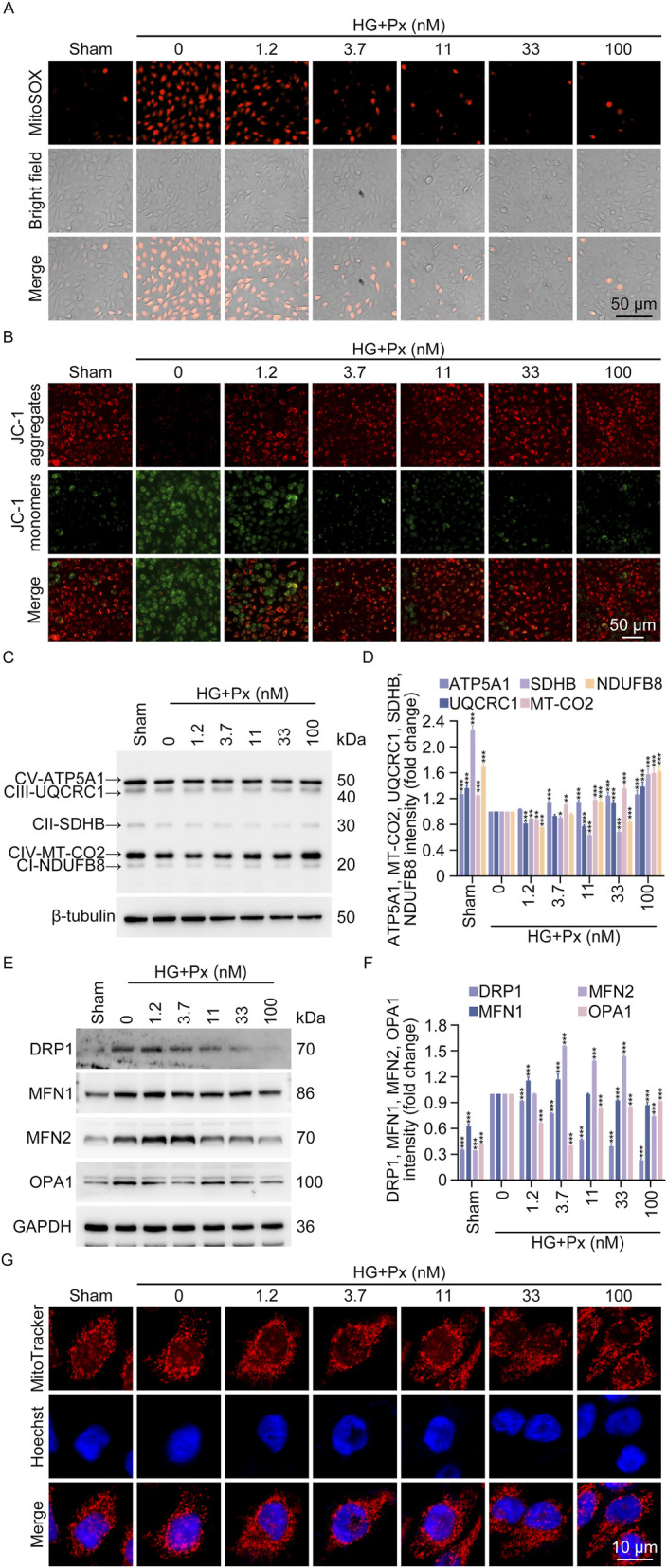
Piroxicam alleviated mitochondrial oxidative stress and restored the mitochondrial function of HG-induced HaCaT cells. After 24 h of induction with HG (60 mM), HaCaT cells were treated with piroxicam (0, 1.2, 3.7, 11, 33, 100 nM) for 24 h. **(A)** The level of ROS within mitochondria was assessed using MitoSOX staining, and red fluorescence indicated mitochondrial superoxide (scale bar = 50 μm). **(B)** HaCaT cells were stained with JC-1 dye. Red fluorescence (JC-1 aggregates) indicated healthy mitochondria with a high MMP, while green fluorescence (JC-1 monomers) indicated mitochondrial membrane depolarization (scale bar = 50 μm). **(C)** The expression of mitochondrial respiratory chain complex proteins, including ATP5A1 (complex V), MT-CO2 (complex IV), UQCRC1 (complex III), SDHB (complex II), and NDUFB8 (complex I), of HaCaT cells was determined by Western blotting. **(D)** Quantitative analysis of protein levels of ATP5A1, MT-CO2, UQCRC1, SDHB, and NDUFB8 was performed. **(E)** The expression of mitochondrial dynamics-related proteins, including DRP1, MFN1, MFN2, and OPA1, was measured using Western blotting. **(F)** Quantitative analysis of protein levels of DRP1, MFN1, MFN2, and OPA1 was performed. **(G)** HaCaT cells were stained with MitoTracker and observed using a confocal microscope (scale bar = 10 μm). All data were presented as mean ± SD. *P < 0.05, **P < 0.01, ***P < 0.001 compared to the 0 nM group (*n* = 3). Abbreviations: HG, high glucose; SD, standard deviation; ROS, reactive oxygen species; JC-1, 5,5′,6,6′-tetrachloro-1,1′,3,3′-tetraethylbenzimidazolylcarbocyanine iodide; MMP, mitochondrial membrane potential; ATP5A1, ATP synthase F1 subunit alpha; MT-CO2, mitochondrial-encoded cytochrome c oxidase 2; UQCRC1, ubiquinol-cytochrome c reductase core protein 1; SDHB, succinate dehydrogenase complex iron-sulfur subunit B; NDUFB8, NADH: ubiquinone oxidoreductase subunit B8; DRP1, dynamin-related protein 1; MFN1, mitofusin 1; MFN2, mitofusin 2; OPA1, optic atrophy 1.

Since mitochondria serve as the primary source and target of intracellular ROS, we next examined mtROS and mitochondrial function. As shown in Figure 4A; Supplementary Figure S1, HG significantly enhanced mtROS accumulation, whereas piroxicam mitigated it, especially at concentrations above 3.7 nM. JC-1 staining of HaCaT cells without HG exhibited bright red fluorescence (JC-1 aggregates), indicating polarized mitochondria with high MMP. Exposure to HG resulted in a marked decrease in red fluorescence and a concomitant increase in green fluorescence (JC-1 monomers), reflecting mitochondrial membrane depolarization. Notably, piroxicam dose-dependently restored the red/green fluorescence ratio, indicating protection against HG-induced mitochondrial dysfunction (Figure 4B; Supplementary Figure S1). Moreover, HG significantly decreased the expression of respiratory chain complex proteins in HaCaT cells. Piroxicam significantly abrogated the downregulation of NDUFB8 (complex I), SDHB (complex II), UQCRC1 (complex III), MT-CO2 (complex IV), and ATP5A1 (complex V) of HG-induced HaCaT cells, with the 100 nM piroxicam showing the most pronounced effect (Figures 4C,D). As shown in Figures 4E,F, HG significantly increased the expression of the fission protein DRP1 as well as the fusion proteins MFN1, MFN2, and OPA1. This concurrent upregulation of both fission and fusion proteins was atypical, as hyperglycemia is generally associated with reduced fusion. It suggested a profound disturbance of mitochondrial dynamics, potentially with DRP1 driving excessive fragmentation, while the elevated fusion proteins may represent a compensatory stress response. Notably, piroxicam treatment restored all these changes toward control levels. The morphological changes of mitochondria were further detected by MitoTracker staining. As shown in Figure 4G, mitochondria in the Sham group exhibited elongated tubular structures uniformly distributed throughout the cytoplasm, forming an intact, interconnected mitochondrial network, indicating a steady-state equilibrium between fission and fusion. However, mitochondria of the 0 nM group exhibited marked fragmentation, pronounced swelling, and a punctate distribution clustered around the nucleus, indicating disrupted fission-fusion dynamics leading to morphological abnormalities. Following piroxicam treatment, mitochondria regained normal morphology with more uniform distribution, demonstrating a dose-dependent recovery.

**FIGURE 5:**
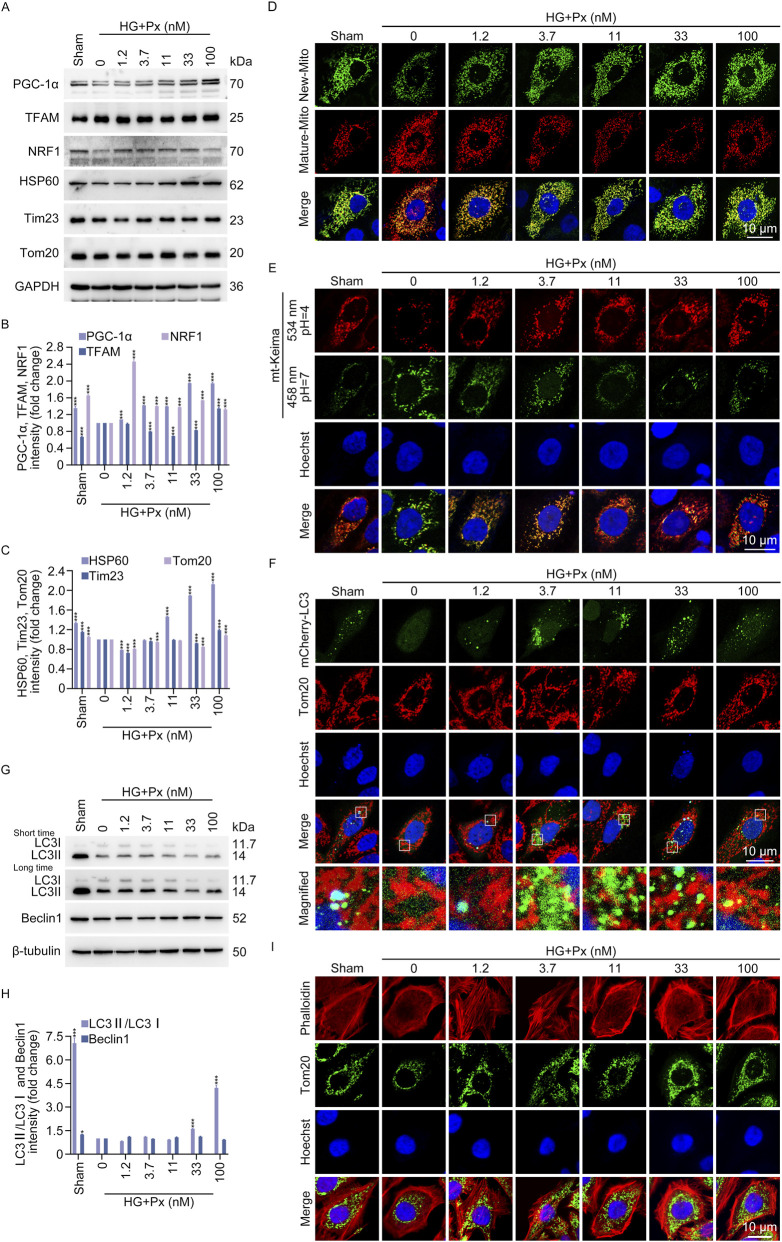
Piroxicam regulated mitochondrial quality control in HG-induced HaCaT cells to maintain mitochondrial function. HG-induced HaCaT cells were treated with Piroxicam (0, 1.2, 3.7, 11, 33, 100 nM) for 24 h. **(A)** The expression levels of key mitochondrial biogenesis proteins, including PGC-1α, TFAM, and NRF1, were measured by Western blotting. Concurrently, key proteins in mitochondrial quantity control were also assessed, including HSP60, the inner mitochondrial membrane translocase Tim23, and the outer mitochondrial membrane translocase Tom20. **(B)** Quantitative analysis of the protein levels of PGC-1α, TFAM, and NRF1 was performed using ImageJ. **(C)** Quantitative analysis of the protein levels of HSP60, Tim23, and Tom20 was performed using ImageJ. **(D)** HaCaT cells stably expressing MitoTimer were imaged using confocal microscopy to monitor mitochondrial turnover. New-Mito emits green fluorescence, while Mature-Mito emits red fluorescence (scale bar = 10 μm). **(E)** HaCaT cells stably expressing mt-Keima were imaged using confocal microscopy to assess mitophagy. The green fluorescence (458 nm) indicated mitochondria in the cytoplasm, while the red fluorescence (534 nm) indicated mitochondria in autolysosomes (scale bar = 10 μm). **(F)** HaCaT cells stably expressing mCherry-GFP-LC3 were induced with HG and treated with piroxicam. Then, the cells were incubated with an anti-Tom20 antibody and imaged using a confocal microscope (scale bar = 10 μm). **(G)** Western blotting was performed to detect the expression of mitophagy-related proteins LC3 and Beclin 1. **(H)** Quantitative analysis of the ratio of LC3II to LC3I and the expression of Beclin1 was performed using ImageJ. **(I)** Representative images showing co-localization of microfilaments (phalloidin) and mitochondria (Tom20) in HaCaT cells (scale bar = 10 μm). All data were presented as mean ± SD. *P < 0.05, **P < 0.01, ***P < 0.001 compared to the 0 nM group (*n* = 3). Abbreviations: HG, high glucose; SD, standard deviation; PGC-1α, peroxisome proliferator-activated receptor gamma coactivator 1-alpha; TFAM, mitochondrial transcription factor A; NRF1, nuclear respiratory factor 1; HSP60, heat shock protein 60; MitoTimer, mitochondrial-targeted Timer; mt-Keima, mitochondrial-targeted Keima; LC3, microtubule-associated protein 1A/1B-light chain 3.

HG-induced mitochondrial morphological abnormalities may result from impaired biogenesis and mislocalization. To further investigate the regulatory mechanisms of piroxicam, we examined markers of mitochondrial biogenesis. As shown in Figures 5A,B, piroxicam reversed the HG-induced downregulation of PGC-1α, NRF1, and TFAM, restoring mitochondrial regenerative capacity. HG consistently disrupted mitochondrial proteostasis by suppressing the translocase of the outer membrane (TOM)/translocase of the inner membrane (TIM) machinery (Tom20, Tim23), as well as the matrix chaperone HSP60, leading to impaired respiratory complex assembly and mitochondrial dysfunction (Figures 5A,C). Piroxicam restored this protein homeostasis network, facilitating proper mitochondrial localization and function. As detected by MitoTimer staining, HG resulted in a significant shift toward red fluorescence (Mature-Mito), accompanied by a reduction in green fluorescence (New-Mito), indicating oxidative stress and accumulation of damaged or senescent mitochondria. Importantly, piroxicam reversed the fluorescence shift, restoring the green-dominant phenotype (Figure 5D; Supplementary Figure S1). Given the pivotal role of mitophagy in mitochondrial turnover, mt-Keima was used to monitor mitophagy. As shown in Figure 5E; Supplementary Figure S1, cells of the Sham group exhibited a mixture of green (mitochondrial, 458 nm) and red (lysosomal mitochondrial, 534 nm) fluorescence, indicating basal mitophagic activity. HG markedly decreased red puncta and increased green puncta, suggesting suppression of mitophagy and accumulation of mitochondria in the cytosol. Piroxicam dose-dependently reversed this shift, restoring red fluorescence and indicating restoration of mitophagic clearance, with the highest mitophagic level at 33 nM. Piroxicam’s effects on mitophagy were further confirmed by evaluating LC3 and Tom20 colocalization. As shown in Figure 5F; Supplementary Figure S1, HG induced mitochondria fragmentation, as indicated by the short, rod-shaped morphology of Tom20-labeled mitochondria. This was accompanied by a near absence of LC3 puncta and a significant loss of LC3-Tom20 colocalization, reflecting a block in mitophagy. Piroxicam effectively reversed this phenotype, restoring both LC3 puncta formation and its colocalization with mitochondria. To assess autophagic activity, we examined LC3 conversion and Beclin1 expression by Western blot. As shown in Figures 5G,H, HG markedly suppressed LC3I to LC3II conversion, evidenced by a decreased LC3II/LC3I ratio and reduced Beclin1 levels, indicating impaired autophagosome nucleation. Notably, piroxicam reversed these alterations, restoring both the LC3II/LC3I ratio and Beclin1 expression, especially at 11 nM. Furthermore, to visualize mitochondrial distribution and their interaction with the actin cytoskeleton, cells were co-stained with Tom20 (mitochondria) and phalloidin (microfilaments). Under HG, mitochondria exhibited fragmentation and perinuclear aggregation, accompanied by blurred microfilament boundaries and their disengagement from mitochondria. Piroxicam restored cytoskeleton integrity, mitigated mitochondrial fragmentation, and normalized the interaction between mitochondria and microfilaments, as evidenced by the restoration of their colocalization signal (Figure 5I).

### 3.5 Piroxicam ameliorated HG-triggered keratinocyte damage and rescued intracellular homeostasis, an advantageous pharmacological effect that was less prominent upon treatment with the selective COX-2 inhibitor celecoxib

**FIGURE 6:**
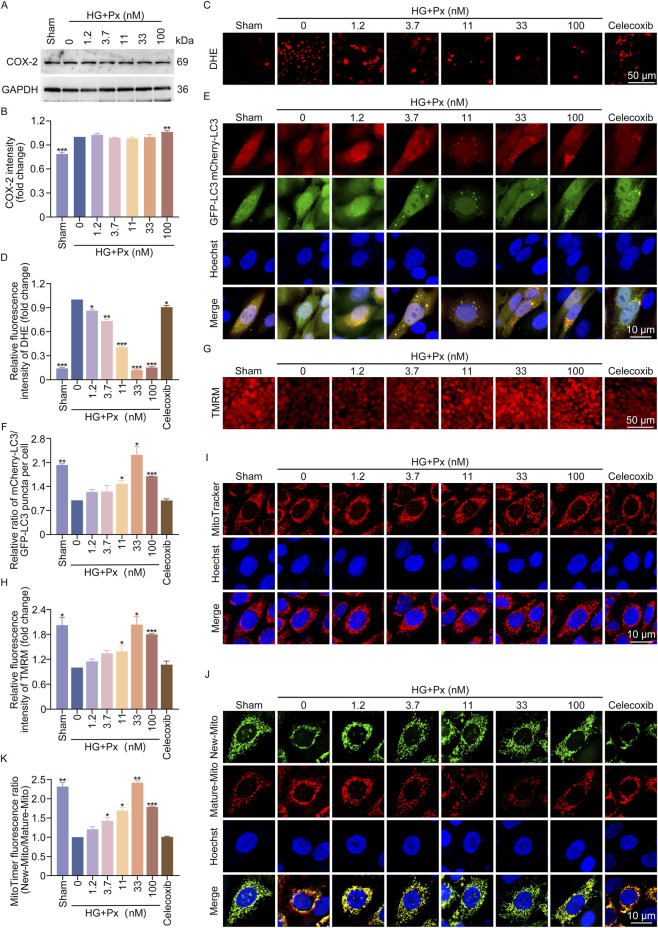
Effects of piroxicam and celecoxib on COX-2 expression, oxidative stress, autophagy, and mitochondrial function in HG-treated HaCaT cells. HG-stimulated HaCaT cells were first incubated for 24 h and then treated with different concentrations of piroxicam (0, 1.2, 3.7, 11, 33, 100 nM) or celecoxib (1 μM) for an additional 24 h. **(A)** Western blotting was performed to detect COX-2 protein expression. GAPDH was used as the loading control. **(B)** Quantitative analysis of COX-2 protein expression. **(C)** Intracellular ROS levels were assessed by DHE staining (scale bar = 50 μm). **(D)** Quantitative analysis of DHE fluorescence intensity. **(E)** Representative confocal images of mCherry-GFP-LC3 to assess autophagic flux (scale bar = 10 μm). **(F)** Quantitative analysis of autophagic flux (mCherry/GFP ratio). **(G)** Mitochondrial membrane potential (MMP) was evaluated using TMRM staining (scale bar = 50 μm). **(H)** Quantitative analysis of TMRM fluorescence intensity. **(I)** MitoTracker staining was used to observe mitochondrial morphology. MitoTracker (red), Hoechst (blue) (scale bar = 10 μm). **(J)** MitoTimer stable-expressing cells were used to assess mitochondrial turnover: New-Mito (newly formed mitochondria, green) and Mature-Mito (aged mitochondria, red). Hoechst (blue) (scale bar = 10 μm). **(K)** Quantitative analysis of the New-Mito/Mature-Mito fluorescence ratio of MitoTimer. All data were presented as mean ± SD. *P < 0.05*,* **P < 0.01*,* ***P < 0.001 compared to the 0 nM group (*n* = 3)*.* Abbreviations: HG, high glucose; Px, Piroxicam; COX-2, cyclooxygenase-2; DHE, dihydroethidium; ROS, reactive oxygen species; LC3, microtubule-associated protein 1A/1B-light chain 3; TMRM, tetramethylrhodamine methyl ester; MMP, mitochondrial membrane potential; MitoTimer, mitochondrial-targeted Timer.

To further investigate whether the protective effect of piroxicam against HG-induced keratinocyte damage stems from COX-2 inhibition, we first examined COX-2 expression levels. Western blot results showed that HG stimulation significantly upregulated COX-2 protein expression in HaCaT cells, whereas piroxicam did not significantly change COX-2 protein levels (Figures 6A,B).

Subsequently, we employed the selective COX-2 inhibitor celecoxib as a control for further evaluation. DHE staining showed that HG significantly increased intracellular ROS levels, whereas piroxicam reduced ROS levels in a dose-dependent manner. Celecoxib also reduced ROS accumulation, whereas piroxicam (3.7, 11, 33, 100 nM) exerted a more pronounced effect (Figures 6C,D). As shown in Figures 6E,F, HG obviously impaired autophagic flux, as evidenced by a significant decrease in the mCherry-LC3/GFP-LC3 spot ratio. Following piroxicam treatment, this ratio increased significantly, indicating that autophagic flux was restored. Treatment with celecoxib showed a similar trend, but the change in fluorescence ratio was negligible.

Consistent with our previous data, TMRM staining showed that HG significantly reduced the MMP, suggesting impaired mitochondrial function. Piroxicam significantly restored MMP levels, and a certain protective effect was also observed following celecoxib treatment (Figures 6G,H). MitoTracker staining further revealed that under HG, mitochondria exhibited marked fragmentation and a disrupted mitochondrial network. Piroxicam significantly improved mitochondrial network integrity and restored a more elongated mitochondrial morphology, while the celecoxib group also showed a similar but relatively less pronounced trend of improvement (Figure 6I). Additionally, MitoTimer analysis (where green fluorescence represents New-Mito and red fluorescence represents Mature-Mito) revealed that HG increased the accumulation of senescent mitochondria. Following treatment with piroxicam, the green-to-red fluorescence ratio in MitoTimer staining images significantly increased, suggesting improved mitochondrial quality, while celecoxib induced relatively mild changes in the fluorescence ratio (Figures 6J,K). So, piroxicam effectively alleviated HG-induced oxidative stress, restored autophagy, improved mitochondrial membrane potential and mitochondrial network integrity, and enhanced mitochondrial quality control. The selective COX-2 inhibitor celecoxib also showed some protective effects on these parameters, but to a lesser extent than piroxicam.

### 3.6 Piroxicam partially attenuated DFU healing and mitochondrial dysfunction in OVX mice, suggesting an ERα-dependent mechanism

**FIGURE 7:**
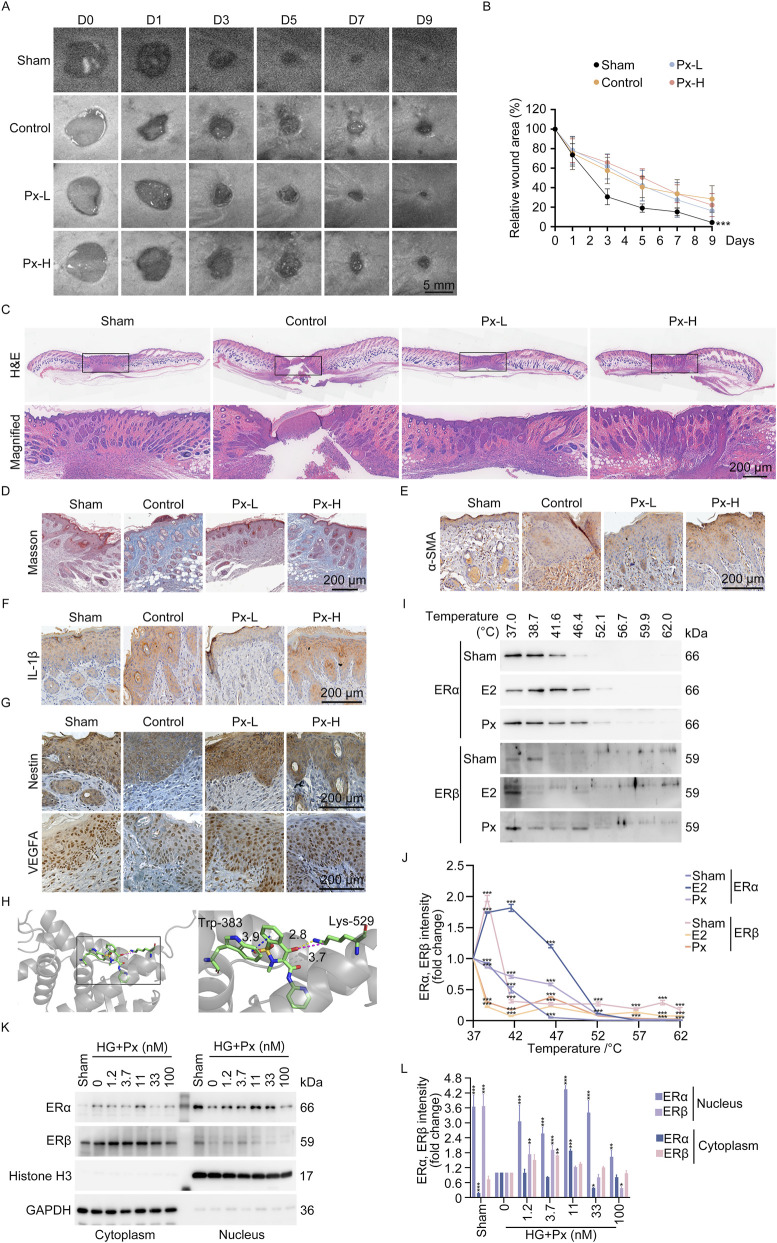
Piroxicam promoted DFU healing by binding to ERα. **(A)** OVX female C57BL/6 J mice were used to establish a DFU murine model via intraperitoneal STZ (50 mg/kg) injections combined with a high-fat diet. Mice with random fasting blood glucose levels ≥ 11.3 mmol/L were included in the study. Full-thickness circular wounds (6 mm diameter) were created on the dorsal skin using a biopsy punch, and the day of wounding was designated as Day 0. Piroxicam (1%, 0.33%) solution or saline was applied to the Px-H, Px-L, Control, and Sham groups, respectively, for 9 consecutive days. Representative images of the wounds on day 0, 1, 3, 5, 7, and 9 were shown (scale bar = 5 mm). **(B)** The relative wound area was quantified using ImageJ and calculated using the following formula: (A_0_ - A_t_)/A_0_ × 100%, where A_0_ represents the initial wound area and A_t_ represents the unhealed wound area on days 0, 1, 3, 5, 7, and 9. **(C)** The skin tissues around wounds of female mice on day 9 post-puncture were analyzed using H&E staining (scale bar = 200 μm). **(D)** Representative images of Masson’s trichrome staining of the dorsal wound skin of female mice on day 9 (scale bar = 200 μm). **(E–G)** Representative images of immunohistochemical staining of α-SMA, IL-1β, Nestin and VEGFA in skin tissues around the wound of female mice (scale bar = 200 μm). **(H)** The ERα LBD was depicted as a gray cartoon model, while piroxicam was shown as a stick model with carbon atoms in green, nitrogen atoms in blue, oxygen atoms in red, and sulfur atoms in yellow. Piroxicam was stably embedded within the hydrophobic pocket of the ERα LBD. A π–π stacking interaction formed between the benzene ring of the piroxicam molecule and the indole ring of Trp-383, with a distance of 3.9 Å (indicated by the blue dashed line). The hydroxyl group on the nitrogen-containing and sulfur-containing six-membered heterocycle of the piroxicam molecule formed two hydrogen bonds with the amino group of the Lys-529 side chain, at interaction distances of 2.8 Å and 3.7 Å (indicated by yellow and purple dashed lines, respectively). **(I)** The binding of piroxicam and ERα was evaluated by CETSA. Briefly, HEK293T cells were treated with piroxicam (33 nM) and E2 (10 nM) for 8 h, and then incubated at eight temperature gradients for 4 min. Subsequently, the protein levels of ERα and ERβ in the cells were determined using Western blotting and quantified with ImageJ **(J)**. **(K)** HG-induced HaCaT cells were treated with Piroxicam (0, 1.2, 3.7, 11, 33, 100 nM) for 24 h. Cytoplasmic and nuclear fractions were isolated, and ERα and ERβ expression levels were assessed using Western blotting. **(L)** Quantitative analysis of ERα and ERβ levels in the nucleus and cytoplasm was performed. All data were presented as mean ± SD. *P < 0.05, **P < 0.01, ***P < 0.001 compared to the Control or 0 nM group (*n* = 3). Abbreviations: HG, high glucose; SD, standard deviation; OVX, ovariectomy; STZ, streptozotocin; Px-L, low-dose piroxicam; Px-H, high-dose piroxicam; H&E, hematoxylin and eosin; α-SMA, alpha-smooth muscle actin; IL-1β, interleukin-1β; Nestin, neuroepithelial stem cell protein; VEGFA, vascular endothelial growth factor A; ERα, estrogen receptor alpha; LBD, ligand-binding domain; Lys-529, lysine-529; Trp-383, tryptophan-383; ERβ, estrogen receptor beta; CETSA, cellular thermal shift assay.

The above results suggest that mechanisms beyond COX-2 inhibition contribute to piroxicam’s protective effects. To further determine whether estrogen signaling mediates these effects *in vivo*, we utilized an OVX murine DFU model. As shown in Figures 7A,B, consistent with the results in male mice, the Sham group achieved nearly complete healing by day 9, characterized by rapid contraction, neat margins, and intact scab formation. In contrast, the Control group exhibited significantly inhibited wound healing, characterized by delayed closure (*P < 0.001*), persistent large wound areas, blurred margins, and sparse granulation tissue. Mice in the Px-H group showed no significant differences in healing compared to the Control group, with persistent exudate and minimal granulation tissue. Only low-dose piroxicam decreased the relative wound area and accelerated the healing. Although this was not statistically significant compared to the Control group, low-dose piroxicam did show a trend promoting the healing. However, because of a deficiency in endogenous estrogen, the efficacy of piroxicam may not have reached the levels observed in male mice. Consistently, H&E staining (Figure 7C; Supplementary Figure S2) showed that low-dose piroxicam improved wound histopathology and reduced inflammatory infiltration. Quantitative analysis of epidermal thickness confirmed that piroxicam promoted re-epithelialization compared to the Control group. Masson’s trichrome staining (Figure 7D; Supplementary Figure S2) showed that low-dose piroxicam, but not high-dose piroxicam, improved collagen organization and increased collagen deposition. Furthermore, as shown in Figures 7E,F, immunohistochemical staining of α-SMA and IL-1β revealed that mice of the Control and Px-H groups both exhibited increased brownish-yellow deposits in the basal layer, indicating exacerbated fibrosis and inflammation. Conversely, the Px-L group showed decreased α-SMA and IL-1β expression compared to the Control group, although levels remained slightly higher than those of the Sham group. Similarly, we further examined the expression of the angiogenesis-related markers Nestin and VEGFA using immunohistochemistry (Figure 7G). Compared with the Sham group, the Control group showed significantly reduced Nestin and VEGFA positivity in wound tissue. Following treatment with low-dose piroxicam, the expression levels of both markers increased significantly, but did not return to the levels observed in the Sham group. The results indicate that piroxicam can partially improve the impaired angiogenesis process under estrogen-deprived conditions. The diminished efficacy of piroxicam in OVX female mice suggested that its therapeutic action may involve mimicking estrogen by directly binding to estrogen receptors.

To further elucidate the target of piroxicam, molecular docking was subsequently employed to predict the binding affinity and site between piroxicam and ERα, which is the most ubiquitously expressed and well-characterized estrogen receptor subtype and a member of the nuclear receptor superfamily mediating the classical genomic actions of estrogen in various tissues, including the skin and its appendages. As shown in Figure 7H, piroxicam was precisely embedded into the key hydrophobic pocket of the ERα ligand-binding domain. The benzene ring structure of the piroxicam molecule formed a π-π stacking interaction with the indole ring of ERα′s tryptophan at position 383 (Trp-383), with an interatomic distance of 3.9 Å. The hydroxyl group on piroxicam’s nitrogen- and sulfur-containing six-membered heterocycle formed two hydrogen bonds with the amino side chain of ERα lysine 529 (Lys-529), with bond distances of 2.8 Å and 3.7 Å, respectively. These results indicated strong interactions between piroxicam and ERα, suggesting piroxicam may directly bind to ERα. The direct binding between piroxicam and ERα was further assessed using CETSA. As shown in Figures 7I,J, piroxicam bound to both ERα and ERβ, thereby enhancing their thermal stability. Under identical temperature conditions, ERα protein expression levels were significantly higher than ERβ, suggesting that the piroxicam-ERα binding is more stable than piroxicam-ERβ. To further validate ERα as the primary receptor for piroxicam rather than ERβ, nuclear-cytoplasmic fractionation of HaCaT cells was determined using Western blotting. As shown in Figures 7K,L, HG induced a marked decrease in nuclear ERα protein levels, which was reversed by piroxicam, especially at 11 and 33 nM, although the 100 nM group still exhibited relatively low nuclear ERα levels. Additionally, piroxicam did not increase ERβ levels at any dose. These results suggested that piroxicam promoted the nuclear translocation of ERα, not ERβ.

**FIGURE 8:**
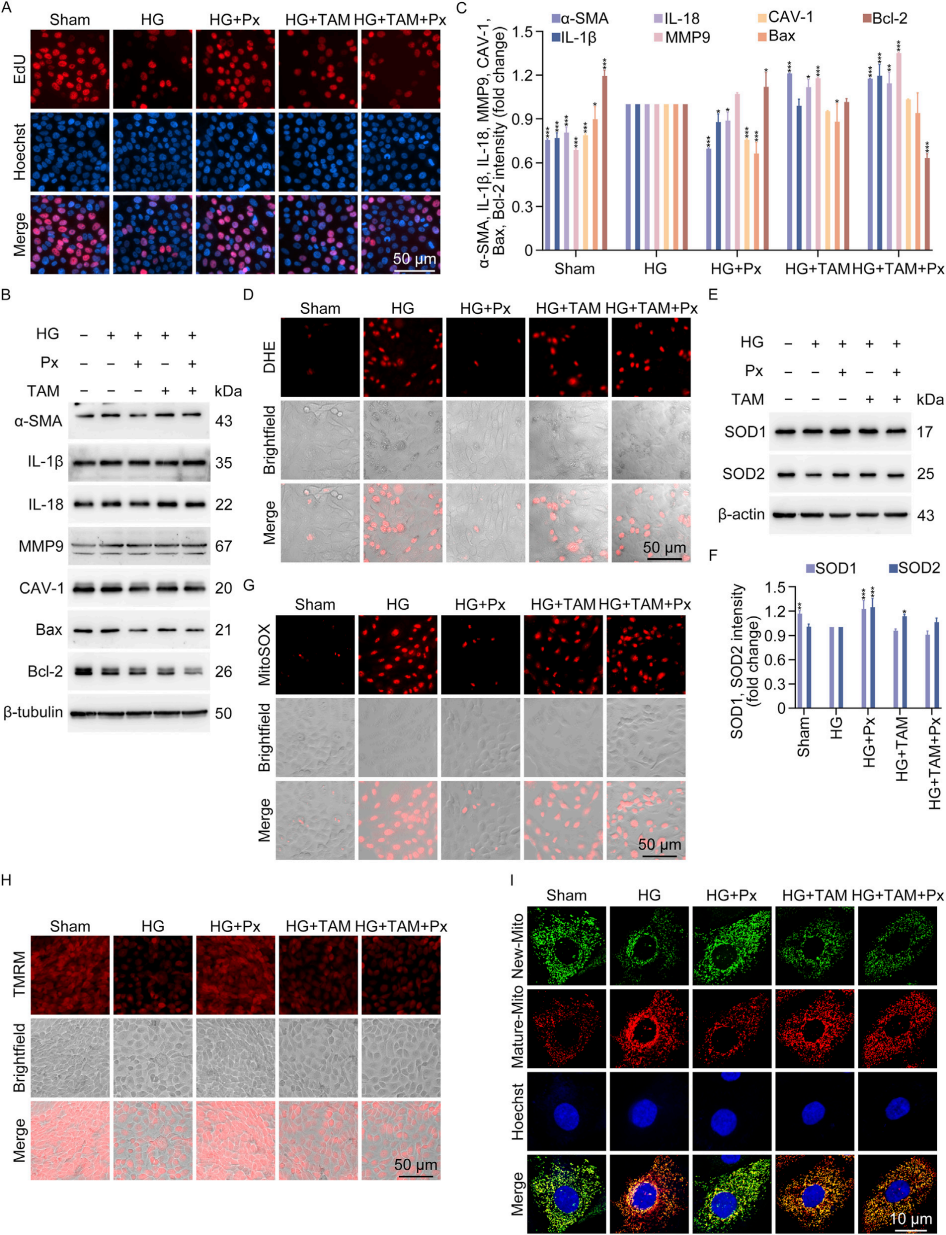
Piroxicam ameliorated HG-induced suppression of proliferation, mitochondrial dysfunction, and oxidative stress via ERα. HaCaT cells were induced with HG (60 mM) for 24 h, followed by incubation with TAM (15 μM) or piroxicam (33 nM) for 24 h. **(A)** Representative images of EdU staining (scale bar = 50 μm). **(B)** Western blotting was employed to detect the expression of α-SMA, MMP9, IL-1β, IL-18, CAV-1, Bax, and Bcl-2. **(C)** Quantitative analysis of protein levels of α-SMA, MMP9, IL-1β, IL-18, CAV-1, Bax, and Bcl-2 was performed using ImageJ. **(D)** Intracellular ROS was detected using DHE staining (scale bar = 50 μm). **(E)** Western blotting was used to assess the expression of the oxidative stress-related proteins, SOD1 and SOD2. **(F)** Quantitative analysis of the protein levels of SOD1 and SOD2 was performed using ImageJ. **(G)** HaCaT cells were stained with the mitochondrial superoxide indicator MitoSOX (scale bar = 50 μm). **(H)** HaCaT cells were incubated with a TMRM probe for 30 min (scale bar = 50 μm) to detect MMP. **(I)** HaCaT cells were infected with MitoTimer lentivirus. Then, the cells were fixed, permeabilized, and stained with Hoechst to label nuclei, and New-Mito (green) and Mature-Mito (red) were visualized (scale bar = 10 μm). All data were presented as mean ± SD. *P < 0.05, **P < 0.01*,* ***P < 0.001 compared to the HG group (*n* = 3). Abbreviations: HG, high glucose; SD, standard deviation; ERα, estrogen receptor alpha; TAM, tamoxifen; α-SMA, alpha-smooth muscle actin; MMP9, matrix metalloproteinase-9; IL-1β, interleukin-1β; IL-18, interleukin-18; CAV-1, caveolin-1; Bax, BCL2-associated X protein; Bcl-2, B-cell lymphoma 2; DHE, dihydroethidium; ROS, reactive oxygen species; SOD1, superoxide dismutase 1; SOD2, superoxide dismutase 2; TMRM, tetramethylrhodamine methyl ester; MMP, mitochondrial membrane potential; MitoTimer, mitochondrial-targeted Timer.

Additionally, the role of ERα in piroxicam’s efficacy was validated using TAM, an estrogen receptor antagonist. EdU staining results revealed that HG significantly decreased the number of EdU-positive cells, indicating suppressed proliferation (Figure 8A; Supplementary Figure S2). This suppression was mitigated by piroxicam. However, the protection of piroxicam was abolished in the presence of TAM. Cells treated with HG in combination with TAM, as well as HG in combination with TAM and piroxicam, continued to exhibit an obvious reduction in EdU-positive signals. As shown in Figures 8B,C, piroxicam significantly reduced the HG-induced upregulation of α-SMA, MMP9, CAV-1, IL-1β, IL-18, and Bax, while increasing the HG-induced downregulation of Bcl-2. TAM blocked the rescue effects of piroxicam on fibrosis, inflammation, and apoptosis. As revealed by DHE staining, piroxicam significantly decreased the red fluorescence induced by HG, which was diminished by TAM (Figure 8D; Supplementary Figure S2). Consistently, piroxicam markedly reduced the HG-induced downregulation of antioxidant proteins SOD1 and SOD2, which was also reversed by TAM (Figures 8E,F). These results suggested that the suppression on intracellular ROS and enhanced antioxidant capacity of piroxicam were abolished by TAM. Additionally, as evidenced by MitoSOX staining, piroxicam significantly reduced mtROS levels, indicating decreased mitochondrial oxidative stress. Following ERα blockade by TAM, piroxicam failed to effectively alleviate mitochondrial oxidative stress (Figure 8G; Supplementary Figure S2). As shown in Figure 8H; Supplementary Figure S2, as detected by TMRM staining, piroxicam enhanced red fluorescence intensity, indicating that piroxicam increased MMP in HG-induced HaCaT cells. Following TAM blockade, piroxicam failed to enhance intracellular red fluorescence intensity, indicating that it did not increase MMP. Furthermore, as detected by MitoTimer staining (green: New-Mito; red: Mature-Mito), piroxicam reversed the HG-induced red-dominant phenotype, restoring the green-dominant phenotype, which was also abrogated by TAM (Figure 8I; Supplementary Figure S2). These findings suggested that piroxicam, as a potential ERα ligand, directly binds to ERα to enhance proliferation, inhibit inflammation, alleviate oxidative stress, and restore mitochondrial function.

## 4 Discussion

DFU remained a severe clinical challenge worldwide due to high morbidity, substantial recurrence, and poor prognosis (Armstrong et al., 2023). Currently available treatments are limited by multiple factors, including insufficient efficacy, significant individual variation, stringent patient selection criteria, and high equipment requirements (OuYang et al., 2024; Smith and Rai, 2024). The substantial unmet needs underscore the importance of exploring alternative therapeutic strategies and identifying novel druggable targets. This study thoroughly investigated the pro-healing effects of piroxicam with DFU models established on male and OVX female mice. The mechanisms of piroxicam were elucidated by employing HG-induced HaCaT cells. Piroxicam promoted DFU healing by stabilizing mitochondrial respiratory chain function, regulating mitochondrial biogenesis, and enhancing mitophagy. Piroxicam also alleviated oxidative stress, suppressed the NF-κB/NLRP3 inflammatory signaling pathway, and inhibited the release of inflammatory cytokines, including IL-1β and IL-18. To investigate whether these protective effects of piroxicam depend on the classical COX-2 inhibition mechanism, we used the selective COX-2 inhibitor celecoxib as a control. Although celecoxib also exhibited some protective effects in certain parameters, it failed to fully replicate the overall biological effects observed with piroxicam. These results suggest that the beneficial effects of piroxicam may not be entirely attributable to COX-2 inhibition, but may also involve other molecular mechanisms. Furthermore, this study revealed that piroxicam exhibited estrogen-mimicking effects in castrated female mice, acting through directly binding to ERα. Upon blocking ERα by tamoxifen, piroxicam’s protection against HG-induced mitochondrial dysfunction, oxidative stress, and inflammation was all abolished.

Our study revealed the promising efficacy of piroxicam in promoting DFU healing, as evidenced by *in vivo* and *in vitro* results. Although piroxicam has never been investigated in DFU, its effects in diabetes or other diabetic complications have been extensively reported. A study revealed that piroxicam can significantly delay the decline in the amplitude of the sensory nerve action potential in diabetic rats, suggesting that it may inhibit the progression of diabetic neuropathy by inhibiting platelet aggregation (Parry and Kozu, 1990). Piroxicam was reported to reduce the incidence of cataracts from 81.8% to 59.1% in diabetic rats, showing a protective effect against diabetic lens opacification (Bahgat et al., 1991). A study combining computer simulation with *in vitro* fluorescence spectroscopy screened and verified that piroxicam has DPP-4 inhibitory activity and proposed that it could be used as a drug-repurposing candidate for the treatment of diabetes (Chittepu et al., 2019). A clinical trial enrolling 75 patients with diabetic neuropathy using a step-by-step treatment protocol based on pain classification, in which piroxicam combined with the muscle relaxant methocarbamol was specifically used to treat muscle pain, resulted in a significant improvement in pain in 66% of patients, confirming the effectiveness of this protocol (Pfeifer et al., 1993). A study using a formalin-induced pain model found that piroxicam was effective in both acute and chronic pain in diabetic rats, whereas diclofenac and ketorolac were ineffective, suggesting that piroxicam has greater analgesic efficacy in diabetes (Alimoradian et al., 2021). Notably, members of the SNAIL family have been shown to regulate keratinocyte activation and re-epithelialization during wound healing (Arnoux et al., 2008), suggesting that the altered SNAIL1 expression observed in this study may reflect wound repair-associated epithelial remodeling rather than a classical fibrotic EMT process. Our study demonstrated that piroxicam significantly enhanced delayed repair in diabetic mice by enhancing keratinocyte proliferation, re-epithelialization, and angiogenesis, inhibiting cellular senescence, and regulating collagen deposition and fibrosis. These reports and results underscored the substantial potential of piroxicam for DFU. As a potent non-steroidal anti-inflammatory drug, piroxicam has well-documented anti-inflammatory effects (Brogden et al., 1981). A study employing the cotton ball granuloma model in alloxan-induced diabetic rats observed that the anti-inflammatory response to piroxicam was significantly weakened and almost completely failed when combined with adrenalectomy, suggesting that the diabetic state may attenuate its anti-inflammatory effects by interfering with the neuroendocrine axis (Valle et al., 1985). In contrast, our study observed obvious anti-inflammatory effects of piroxicam in the skin of STZ and HFD-induced mice, suggesting that its efficacy may vary depending on the type of inflammation, the course of diabetes, organs, or neuroendocrine functional integrity. Due to the low immune response in the early phase of DFU, local infection cannot be cleared, and it evolves into a chronic, non-resolving inflammatory environment characterized by a continuous increase in MMPs/ROS/pro-inflammatory factors. This microenvironment is the key difference between DFU and ordinary wounds (Mohsin et al., 2024; Roy et al., 2024). Our study observed that HG induced significantly dysregulated MMP9 expression, boosted ROS production, and increased the release of pro-inflammatory cytokines such as IL-1β and IL-18. These results supported that non-resolving inflammation is the core pathological mechanism of DFU (Dong et al., 2024). These changes were reversed by piroxicam, supporting piroxicam’s efficacy against a non-resolving inflammatory microenvironment. Interestingly, SOD2 expression levels decreased following piroxicam treatment. Given that SOD2 is often upregulated as a compensatory response to oxidative stress, this reduction may indicate that oxidative stress has been alleviated and the need for antioxidant compensation has diminished. Chronic wounds, such as venous leg ulcers and pressure ulcers, share a core pathological mechanism of dysregulated immunity and persistent inflammation, characterized by macrophage dysfunction, prolonged release of pro-inflammatory cytokines, and cellular senescence, and often accompanied by biofilm infection (Ma et al., 2025; Riaz et al., 2025). Although there are currently no reports on the effects of piroxicam on these chronic wounds, it holds great promise for them.

Another finding is the identification of a novel mechanism whereby piroxicam alleviates HG-induced mitochondrial dysfunction and restores mitochondrial homeostasis by binding to ERα. Based on current literature, piroxicam exhibits a dual role on mitochondria: in normal tissues such as rat liver/heart mitochondria and hamster fibroblasts, it primarily acts as a damaging agent, functioning as an oxidative phosphorylation uncoupler that disrupts MMP and directly inserts into the mitochondrial membrane, leading to increased permeability and cytochrome c release (Moreno-Sanchez et al., 1999; Chakraborty et al., 2007). However, under disease or pathological conditions, such as in a rat model of colon cancer or in neuronal cells under neurotoxic attack, it instead displays a protective effect on mitochondria, either by modulating the Bcl-2/Bax pathway to induce cancer cell apoptosis or by maintaining energy metabolism in compromised neurons to facilitate rescue (Soliman et al., 2009; Saini et al., 2012). So, piroxicam acts as a double-edged sword, with its ultimate effect on mitochondria being highly dependent on the specific cellular context and pathological environment. Consistently, our study revealed that piroxicam increased mitochondrial biogenesis, inhibited mtROS, enhanced mitophagy, and elevated MMP in HG-induced HaCaT cells and DFU mice. It has been reported that ROS-mediated apoptosis was induced in estrogen receptor-positive MCF-7 cells, but not in estrogen receptor-negative MDA-MB-231 cells, suggesting that piroxicam’s effects on mitochondria may be closely related to estrogen receptor (Rai et al., 2015). Especially, activation of ERα maintains mitochondrial homeostasis by regulating Parkin and preserving mitochondrial quality control balance, thereby playing a crucial role in sustaining mitochondrial energy metabolism, biogenesis, and dynamics (Zhou et al., 2025). In the present study, piroxicam significantly promoted diabetic wound healing in male mice, whereas its pro-healing effect was partially attenuated in OVX female mice. Intact female mice were not included because they are relatively resistant to STZ-induced diabetes, making it difficult to establish a stable diabetic wound model (Saadane et al., 2020). Molecular docking indicated that piroxicam, as a potential ERα ligand, achieves specific binding by forming π-π stacking interactions with ERα Trp-383 and hydrogen bonds with Lys-529. This binding promoted nuclear translocation of ERα and activated its downstream signaling pathways. The observation that piroxicam upregulated the ERα transcriptional targets PGC 1α, NRF1, and TFAM (Figures 5A,B), together with the finding that ERα blockade by tamoxifen reduced nascent mitochondria (Figure 8I), supports a model in which piroxicam activates Erα, which drives mitochondrial protection via transcriptional regulation of quality control genes. Consequently, piroxicam partially mimics estrogen’s effects by accelerating wound healing in OVX female mice with DFU. Future studies incorporating estrogen replacement or ERα-specific agonists would help further dissect the specific contribution of ERα signaling to piroxicam’s efficacy. Nuclear-cytoplasmic separation experiments further demonstrated that piroxicam significantly promotes nuclear translocation of ERα, but not ERβ. Moreover, when ERα was blocked by TAM, piroxicam failed to attenuate mitochondrial dysfunction, further confirming ERα′s central role in DFU wound healing and mitochondrial function regulation. We also note that future studies using more selective ERα antagonists, such as fulvestrant, or genetic approaches, would further strengthen the evidence. This study further validated ERα as a key target for mitochondrial-targeted therapy in DFU.

This study revealed that piroxicam exhibits pro-healing effects at low doses, whereas its beneficial effects are diminished or even reversed at high doses in diabetic mice. Consistently, piroxicam’s best efficacy in HG-induced HaCaT cells also appeared at low or moderate doses, not high doses. Besides background dependence, doses further determine the intensity and window of piroxicam’s action. For normal cells, the damaging effects of piroxicam are dose-dependent. Only a slight mitochondrial perturbation was observed at low doses, and cells could survive through self-repair, while mitochondrial membrane permeability was significantly increased and cytochrome c was released in large quantities, eventually triggering irreversible apoptosis (Chakraborty et al., 2007). Similarly, the protection of piroxicam against 1-methyl-4-phenylpyridinium (MPP^+^)-induced toxicity was only achieved within a specific dose range, ineffective below the therapeutic window, and may shift to non-specific toxicity above it (Soliman et al., 2009). In this study, increasing the piroxicam dose did not further enhance wound healing, and no cytotoxicity was observed at higher doses. We initially speculated that high-dose piroxicam might suppress PGE2 levels excessively, but PGE2 levels were below the detection limit in all groups (data not shown), ruling out this explanation. PGE2 levels were generally below detection limits, suggesting weak COX-2 pathway activation in our experiment. This involved HaCaT cells treated with 60 mM glucose for 24 h. The slight protective effect of its selective inhibitor, celecoxib, supports this observation. Instead, the reduced efficacy at the highest dose may reflect a dose-dependent therapeutic window, potentially due to biphasic effects on ERα signaling or off-target inhibition at supra-optimal concentrations, as supported by our *in vitro* dose-response data (e.g., TMRM, mtROS). The precise mechanism warrants further investigation. The 1% piroxicam concentration used here matches commercially available formulations, primarily for local analgesia. However, in mouse experiments, a 0.33% concentration showed better therapeutic effects. This suggests that the widely used clinical concentration may not be optimal for diabetic foot ulcers. Future pharmacokinetic and dose-optimization studies are needed.

Despite this study’s innovative findings, certain limitations remain and require further investigation. We acknowledge the absence of an osmotic control group, such as mannitol or low-concentration glucose, in this study. This limitation precludes the direct exclusion of potential hyperosmotic effects induced by 60 mM glucose. Previous studies using mannitol as an osmotic control have shown that, at this glucose concentration, the cellular effects are primarily due to glucotoxicity rather than hyperosmolarity (Fischereder et al., 2003). Furthermore, 60 mM glucose is widely used to mimic diabetic conditions *in vitro* (Yu et al., 2017; Shi et al., 2022). Nevertheless, we recognize that the lack of a direct osmotic control in our design is a limitation. Future studies incorporating such controls would further strengthen the conclusions. We acknowledge that the 12-h observation window in the scratch assay is relatively short for capturing the full spectrum of ERα-mediated transcriptional events, and future time-course studies with additional time points are warranted. Ideally, direct measurements of mitochondrial respiration, such as oxygen consumption rate via Seahorse analysis, ATP production, and ultrastructural examination by transmission electron microscopy (TEM), are required to fully validate the bioenergetic consequences. The present conclusions, while supported by multiple independent and complementary indirect assays, should be confirmed in future studies using direct functional readouts, such as Seahorse, ATP, and TEM. In addition, although we assessed COX-2 protein expression, we did not measure COX-2 enzymatic activity. Nevertheless, the inclusion of the selective COX-2 inhibitor celecoxib as a pharmacological control supports the conclusion that piroxicam’s protective effects are unlikely to be mediated primarily through the classical COX-2 pathway. Finally, only DFU murine models and HaCaT cells were used, and clinically relevant factors such as infection, the wound microbiome, and long-term safety were not systematically evaluated. Nevertheless, given its established transdermal formulations and well-characterized safety profile, our findings provide a basis for future preclinical and clinical studies. Of course, its efficacy and safety in clinical settings require further validation.

## 5 Conclusion

This study demonstrated that piroxicam promotes DFU healing by improving mitochondrial homeostasis and quality control, including restoring mitochondrial membrane potential, respiratory chain complex levels, and mitophagy, in keratinocytes. Piroxicam also inhibited non-resolving inflammation through suppressing the activation of the cGAS-STING-NF-κB inflammatory pathway. Piroxicam exerts these effects by binding to ERα, promoting its nuclear translocation, and activating downstream signaling pathways. Importantly, low-dose piroxicam showed superior pro-healing efficacy, whereas higher doses were less effective or even harmful, highlighting the importance of dose optimization.

## Data Availability

Data are available from the corresponding author upon reasonable request.

## Supplementary material

SUPPLEMENTARY FIGURE S1 Quantification of histological and immunostaining results in Figures 1–5. **(A)** Quantification of H&E staining shown in Figure 1E. **(B)** Quantification of collagen deposition (Masson’s staining) shown in Figure 1F. **(C)** Quantification of EdU staining shown in Figure 2A. **(D)** Quantification of γ-H2AX immunostaining shown in Figure 2F. **(E)** Quantification of p-ATM immunostaining shown in Figure 2G. **(F)** Quantification of SA-β-gal staining shown in Figure 2H. **(G)** Quantification of DHE staining shown in Figure 3F. **(H)** Quantification of MitoSOX staining shown in Figure 4A. **(I)** Quantification of JC-1 staining shown in Figure 4B. **(J)** Quantification of MitoTimer staining shown in Figure 5D. **(K)** Quantification of mt-Keima staining shown in Figure 5E. **(L)** Quantification of LC3 and Tom20 colocalization shown in Figure 5F. Data were presented as mean ± SD from three independent experiments and normalized to the control group (set as 1). Statistical significance was determined by one-way ANOVA (*P < 0.05, **P < 0.01, ***P < 0.001). Abbreviations: H&E, hematoxylin and eosin; EdU, 5-ethynyl-2′-deoxyuridine; γ-H2AX, phosphorylated histone H2AX (Ser139); p-ATM, phosphorylated ataxia-telangiectasia mutated; SA-β-gal, senescence-associated β-galactosidase; DHE, dihydroethidium; ROS, reactive oxygen species; mtROS, mitochondrial reactive oxygen species; JC-1, 5,5′,6,6′-tetrachloro-1,1′,3,3′-tetraethylbenzimidazolylcarbocyanine iodide; MMP, mitochondrial membrane potential; MitoSOX, mitochondrial superoxide indicator; mt-Keima, mitochondrial-targeted Keima; LC3, microtubule-associated protein 1A/1B-light chain 3; Tom20, translocase of outer mitochondrial membrane 20; SD, standard deviation.

SUPPLEMENTARY FIGURE S2 Quantification of histological and immunostaining results in Figures 7, 8. **(A)** Quantification of H&E staining shown in Figure 7C. **(B)** Quantification of ollagen deposition (Masson’s trichrome staining) shown in Figure 7D. **(C)** Quantification of EdU staining shown in Figure 8A. **(D)** Quantification of DHE staining shown in Figure 8D. **(E)** Quantification of MitoSOX staining shown in Figure 8G. **(F)** Quantification of TMRM immunostaining shown in Figure 8H. **(G)** Quantification of MitoTimer staining shown in Figure 8I. Data were presented as mean ± SD from three independent experiments and normalized to the control group (set as 1). Statistical significance was determined by one-way ANOVA (*P < 0.05, **P < 0.01, ***P < 0.001). Abbreviations: H&E, hematoxylin and eosin; EdU, 5-ethynyl-2′-deoxyuridine; DHE, dihydroethidium; ROS, reactive oxygen species; MitoSOX, mitochondrial superoxide indicator; mtROS, mitochondrial reactive oxygen species; TMRM, tetramethylrhodamine methyl ester; MMP, mitochondrial membrane potential; SD, standard deviation.
